# Tree Diversity Increases Forest Temperature Buffering via Enhancing Canopy Density and Structural Diversity

**DOI:** 10.1111/ele.70096

**Published:** 2025-03-21

**Authors:** Florian Schnabel, Rémy Beugnon, Bo Yang, Ronny Richter, Nico Eisenhauer, Yuanyuan Huang, Xiaojuan Liu, Christian Wirth, Simone Cesarz, Andreas Fichtner, Maria D. Perles‐Garcia, Georg J. A. Hähn, Werner Härdtle, Matthias Kunz, Nadia C. Castro Izaguirre, Pascal A. Niklaus, Goddert von Oheimb, Bernhard Schmid, Stefan Trogisch, Manfred Wendisch, Keping Ma, Helge Bruelheide

**Affiliations:** ^1^ German Centre for Integrative Biodiversity Research (iDiv) Halle‐Jena‐Leipzig Leipzig Germany; ^2^ Systematic Botany and Functional Biodiversity Leipzig University Leipzig Germany; ^3^ Chair of Silviculture, Institute of Forest Sciences University of Freiburg Freiburg Germany; ^4^ Leipzig Institute for Meteorology Leipzig University Leipzig Germany; ^5^ CEFE, Univ Montpellier, CNRS, EPHE, IRD Montpellier Cedex 5 France; ^6^ Jiangxi Key Laboratory of Plant Resources and Biodiversity Jingdezhen University Jingdezhen China; ^7^ Institute of Biology Leipzig University Leipzig Germany; ^8^ Institute of Botany Chinese Academy of Sciences Beijing China; ^9^ Institute of Ecology Leuphana University of Lüneburg Lüneburg Germany; ^10^ Institute of General Ecology and Environmental Protection TUD Dresden University of Technology Tharandt Germany; ^11^ Institute of Biology/Geobotany and Botanical Garden Martin Luther University Halle‐Wittenberg Halle (Saale) Germany; ^12^ Helmholtz Centre Potsdam—GFZ German Research Centre for Geosciences Section 1.4 Remote Sensing and Geoinformatics Potsdam Germany; ^13^ Swiss Federal Institute for Forest, Snow and Landscape Research (WSL) Birmensdorf Switzerland; ^14^ Department of Evolutionary Biology and Environmental Studies University of Zürich Zürich Switzerland; ^15^ Department of Geography, Remote Sensing Laboratories University of Zürich Zürich Switzerland

**Keywords:** BEF‐China, biodiversity, heat stress, microclimate, mixed‐species forest

## Abstract

Global warming is increasing the frequency and intensity of climate extremes. Forests may buffer climate extremes by creating their own attenuated microclimate below their canopy, which maintains forest functioning and biodiversity. However, the effect of tree diversity on temperature buffering in forests is largely unexplored. Here, we show that tree species richness increases forest temperature buffering across temporal scales over six years in a large‐scale tree diversity experiment covering a species richness gradient of 1 to 24 tree species. We found that species richness strengthened the cooling of hot and the insulation against cold daily and monthly air temperatures and temperature extremes. This buffering effect of tree species richness was mediated by enhanced canopy density and structural diversity in species‐rich stands. Safeguarding and planting diverse forests may thus mitigate negative effects of global warming and climate extremes on below‐canopy ecosystem functions and communities.

## Graphical abstract

Making use of 6 years of microclimate measurements in a large‐scale tree diversity experiment covering a species richness gradient ranging from 1 to 24 tree species, we demonstrate that tree species richness significantly enhances forest temperature buffering at daily, monthly, and annual scales.

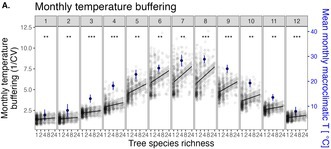

## 1 Introduction

Global warming and its impacts on the world's forests (IPCC 2022) are largely studied as effects of air temperatures measured outside forests in open‐ground conditions (also referred to as macroclimate) (de Frenne et al. 2021). However, this omits that forests can buffer temperature extremes such as hot and cold spells to some extent by creating their own microclimate below their canopy (de Frenne et al. 2019; Geiger et al. 2009), from which other organisms benefit, including sub‐canopy trees. Among earth's terrestrial ecosystems, forests are likely the ones with the strongest air temperature buffering (hereafter ‘temperature buffering’) capacity owing to their often multi‐layered canopies, which provide evapotranspirative cooling and shading, and decrease the mixing of air layers (Zellweger et al. 2020; Geiger et al. 2009). Temperature buffering occurs when microclimate temperature fluctuations are smaller than fluctuations in macroclimate temperatures (de Frenne et al. 2021). Smaller temperature fluctuations below the canopy can be quantified as a lower temporal variance of temperatures, to which in the following we refer to as microclimate temporal stability (Tilman 1999). The differences between macroclimate (outside forest) and microclimate (inside forest) temperatures are substantial, with global averages of −4.1°C ± 0.5°C decreased temperature maxima and 1.1°C ± 0.2°C increased temperature minima below the forest canopy^2^. This difference is larger than the average warming of land surface temperatures in 2011–2020 compared with 1850–1900 (1.3 to 1.8)°C (IPCC 2021).

The temperature buffering capacity of forests has important consequences for forest functioning and biodiversity above‐ and belowground, especially in the context of global warming (de Frenne et al. 2021; Gottschall et al. 2019; Kemppinen et al. 2024). For instance, many physiological processes, such as soil respiration (Chapin III et al. 2011), scale exponentially with temperature, which implies that even small temperature increases may have large effects on rates, underlining the importance of temperature buffering. Furthermore, temperature buffering can influence forest biodiversity by slowing shifts in forest community composition towards warm‐affinity species (i.e., thermophilization) under global warming (Zellweger et al. 2020; de Frenne et al. 2013). However, the reciprocal control of tree diversity on forest temperature buffering remains largely unexplored.

Simulation studies showed that plant diversity can stabilise climate–vegetation feedbacks (Claussen et al. 2013). Moreover, tree species diversity has been shown to increase tree growth in mixtures (Huang et al. 2018; Schnabel et al. 2019) and to enhance canopy complexity (Kunz et al. 2019; Perles‐Garcia et al. 2021), resulting in a greater thickness, density, and structural diversity of the canopy layer (i.e., the buffering layer). It is thus conceivable that tree species richness may increase the temperature buffering capacity of forests by affecting these forest properties. For instance, mean tree height and the area of foliage per unit ground area (i.e., leaf area index; LAI) (Gates and Hanks 1967) as proxies for the thickness and density of the buffering layer modify the energy exchange at the canopy by influencing the penetration of sunlight and its albedo and evapotranspiration, which in turn affects the temperature buffering capacity of the forest (de Frenne et al. 2021). Moreover, structural diversity (McElhinny et al. 2005; Ehbrecht et al. 2021; Aalto et al. 2023) measured, for instance, as stand structural complexity index (SSCI) from terrestrial laser scans (Ehbrecht et al. 2017) may reduce the vertical mixing of air masses (Ehbrecht et al. 2019) and thereby increase temperature buffering. Previous studies provide some evidence that a thick, dense, or structurally diverse tree canopy can promote forest temperature buffering (Ehbrecht et al. 2017, 2019; Donfack et al. 2021; Gillerot et al. 2022; Zellweger et al. 2019; de Frenne et al. 2021). However, it remained unclear if tree species richness supported these forest properties and, thereby, temperature buffering, as former studies rarely investigated the role of tree species richness, and the few reported non‐significant effects (Ehbrecht et al. 2019; Gillerot et al. 2022; Donfack et al. 2021). Hence, there is very little empirical evidence for tree diversity effects on forest temperature buffering in general, and, in particular, regarding the forest properties mediating such diversity–microclimate relationships. Establishing such direct and indirect causal relationships requires studies that experimentally manipulate tree species richness and control for confounding factors, such as environmental variation or species identity effects (Bruelheide et al. 2014; Scherer‐Lorenzen et al. 2005). A pioneering study reported that tree species richness (1‐ vs. 4‐species) increased temperature buffering for some species mixtures (Zhang et al. 2022), but longer diversity gradients and data from multiple years would be necessary to generalise beyond specific species compositions and macroclimatic conditions as well as to understand the mediators of tree diversity effects on temperature buffering and their temporal dynamics.

Tree diversity effects on microclimate temperatures in forests may change over days, months, and years. Compared with open‐ground conditions, temperatures within forests are expected to be higher during night‐time and winter, and lower during day‐time and summer (Gottschall et al. 2019). The underlying reason is that the energy exchange is shifted from the ground surface to the canopy (Stuenzi et al. 2021). Consequently, forest canopies mitigate hot temperatures via evapotranspiration (consumption of latent heat), reflecting or absorbing solar radiation and emitting long‐wave radiation, and insulate against cold temperatures via heat retention (de Frenne et al. 2019; Geiger et al. 2009). However, many more processes may be involved depending on the spatiotemporal scale studied (de Frenne et al. 2021). For example, evapotranspirative cooling effects decrease with decreasing soil water availability (de Frenne et al. 2021; Greiser et al. 2024), highlighting the potential influence of inter‐annual dynamics and extremes in macroclimatic conditions (such as droughts) on temperature buffering. However, the relative importance of tree diversity effects on temperature buffering across temporal scales remains unknown.

Here, we analyse microclimate measurements conducted within forests of 1 to 24 tree species covering six years (2015–2020) from a large‐scale subtropical tree diversity experiment (BEF‐China [Bruelheide et al. 2014; Huang et al. 2018]). Assembling the communities with varying species richness randomly from species pools resulted in stands that differ in canopy thickness (Huang et al. 2018), density (Peng et al. 2017) and structural diversity (Perles‐Garcia et al. 2021). We aim to understand the role of tree species richness and these mediating factors for temperature buffering below forest canopies at different temporal scales (i.e., daily, monthly and yearly). In our subtropical study system, which is characterised by a monsoon climate, high macroclimate temperatures coincide with high water availability for evapotranspirative cooling, which should promote temperature offsets between micro‐ and macroclimate, particularly for maximum temperatures (de Frenne et al. 2021). Hence, we expect tree species richness effects on microclimate to be most pronounced for the buffering of maximum temperatures. We tested the following hypotheses: H1: tree species richness increases the temperature buffering potential of forest canopies via cooling hot and insulating against cold macroclimate temperatures at daily, monthly, and annual time scales. H2: species richness effects on temperature buffering—measured as microclimate temperature stability—are consistently positive across time scales but strongest when macroclimate temperatures are high. H3: positive tree species richness effects on temperature buffering are mediated by enhanced canopy thickness, density, and structural diversity.

## 2 Materials and Methods

### 2.1 Study Site and Experimental Design

We used data from a large‐scale tree diversity experiment, the Biodiversity–Ecosystem Functioning China Experiment (BEF‐China experiment) (Bruelheide et al. 2014), located in Xingangshan, Dexing, Jiangxi (29°08′‐29°11′ N, 117°90′‐117°93′ E; Figure S1). The experiment was established at two sites, A and B, which were planted in 2009 and 2010, respectively. Each site covers approximately 20 ha in size. The site's climate is governed by the subtropical monsoon, with cold and dry winters and hot and humid summers. The mean annual temperature and precipitation are 16.7°C and 1821 mm (mean from 1971–2000) (Yang et al. 2013). Inter‐annual changes in climate‐induced water availability are strong and driven primarily by changes in precipitation and only to a lower degree by changes in temperature (Schnabel et al. 2021). The native forests of the study region harbour a high tree species richness and are dominated by broadleaf tree species (Bruelheide et al. 2014). Based on a total pool of 40 native evergreen and deciduous broadleaf tree species, we created manipulated species richness gradients of 1 to 24 coexisting species (Figure S2; Table S1). Overall, 226,400 individual trees were planted on 566 plots of 25.8 × 25.8 m^2^ with 400 trees per plot. Understorey vegetation, including herbaceous and non‐planted woody species, was removed twice yearly to maintain the desired tree species composition. To increase generality and statistical power, tree species were allocated randomly to extinction scenarios following a broken‐stick design. This design ensures that all species are equally represented at all richness levels by splitting three overlapping 16‐species pools at each site into non‐overlapping species compositions of lower richness (Bruelheide et al. 2014). Moreover, the highest richness level was created through combining 24 species from these pools. Here, we used data from the 64 Very Intensively Studied Plots (VIPs) of the BEF‐China experiment; see Bruelheide et al. (2014) and Huang et al. (2018) for details on the experimental design.

### 2.2 Micro‐ and Macroclimate Measurements

The microclimate air temperature was recorded hourly over six years (January 2015–December 2020) across the VIP plots (32 at each site) using temperature loggers (HOBO Pro v2, U23‐001) covered by a rain‐protection shield and installed at 1 m height in the centre of the plots (see Figures S2–S5). Data were controlled and cleaned to remove unrealistic data due to logger malfunction (e.g., temperature outliers or time series divergent dynamics). Plots with incomplete monthly records were excluded from the monthly analyses, and incomplete yearly records were excluded from yearly analyses (1 plot of the 64 plots was removed in all analyses). Macroclimate data—minimum, average, and maximum monthly temperature (°C), monthly precipitation sum (mm) and monthly potential evapotranspiration (mm) sum—were retrieved from the high‐resolution gridded dataset of the Climatic Research Unit (CRU) Time‐Series (TS) version 4.06 (Harris et al. 2020) with a 0.5° (latitude/longitude) resolution, which is based on interpolated climate station observations. To explore if diversity–microclimate relationships were influenced by water availability, we further calculated the Standardised Precipitation‐Evapotranspiration Index (SPEI) (Vicente‐Serrano et al. 2010) based on these precipitation and evapotranspiration data with the SPEI package (Beguería and Vicente‐Serrano 2017). The SPEI is a commonly used drought index that captures the climatic water balance (precipitation minus potential evapotranspiration) at different time lengths from a single month (SPEI1) to an entire year (SPEI12; January–December). SPEIs below −1 and above 1 can be considered exceptionally dry or wet compared to the average conditions during a climate reference period (McKee et al. 1993) (here 1901–2019).

### 2.3 Temperature Buffering and Stability

Using the hourly microclimate temperature measurements, we calculated different measures describing temperature extremes and temperature buffering. We calculated monthly minimum, median, and maximum microclimate temperatures per plot. Minimum and maximum monthly temperatures were calculated by taking the median of the 5% lowest and 95% highest temperatures, respectively. We quantified temperature buffering on monthly and annual time scales as the temporal stability (Tilman 1999) of microclimate temperature. This stability metric is commonly used in biodiversity–ecosystem functioning studies to provide insights into the stabilising effects of biodiversity for multiple ecosystem processes and at different levels of organisation (Schnabel et al. 2021; Craven et al. 2018; Isbell et al. 2015). Temporal stability (*S*) was calculated as the inverse of the coefficient of variation (CV):

$$S=\frac{\mu }{\sigma }$$

where *μ* and *σ* are the mean and standard deviation of hourly temperature measurements per month or year, hereafter referred to as monthly or annual temperature stability.

### 2.4 Assessment of Microclimate Drivers

We assembled a range of variables describing canopy thickness, density, and structural diversity from former studies and tree inventories in the BEF‐China experiment. Out of these potential variables, we selected the ones with the highest relevance for temperature buffering according to literature‐derived hypotheses (focussing on the ones most successfully used as predictors of temperature buffering in other studies; Method S1; Figure S6; Tables S2, S3) and compared correlations between variables (Figure S7). Specifically, we selected mean tree height, leaf area index (LAI), and Stand Structural Complexity Index (SSCI) to describe canopy thickness, density, and structural diversity, respectively. Tree height was measured as the mean height of the central 6 × 6 trees in each plot to avoid edge effects (Bongers et al. 2021). LAI was measured using digital hemispheric photography at five positions within each plot in August (Peng et al. 2017), and SSCI by a single terrestrial laser scan at the centre of each plot under leaf‐off conditions of the deciduous tree species (February–March) as described by Perles‐Garcia et al. (2021) (see Output S1 for summary statistics). For all forest property variables, we used data collected at site A of the BEF‐China experiment in 2019 (where we had the best data coverage), except for LAI, measured in 2014.

### 2.5 Statistical Analyses

We used linear mixed‐effects models (LMMs) to test for the effects of tree species richness on microclimate temperatures and temperature buffering across time scales and VIP plots (*n* = 63 plots, tree species richness ranging from 1–24 species). We tested for species richness effects on hourly temperatures and on minimum, median, and maximum monthly temperatures using LMMs in which species richness in interaction with hour or month was considered a fixed effect. Similarly, we tested for species richness effects on monthly and annual temperature stability using LMMs in which species richness in interaction with month or calendar year was considered a fixed effect. We accounted for the experimental design of our study through a nested random effect structure of plots nested within the experimental site (A or B) and for temporal autocorrelation by using a first‐order autocorrelation structure (corCAR1) for time covariates (days, months or years). Additionally, we explored how diversity effects, i.e., the slopes of the regressions between species richness and monthly minimum, median, and maximum microclimate temperatures and monthly temperature buffering, depended on macroclimate conditions (monthly minimum, average and maximum temperatures and SPEI values). At the annual scale, we tested if temperature stability was related to annual climatic water balances by replacing calendar years with annual SPEI values in the respective LMM.

**FIGURE 1:**
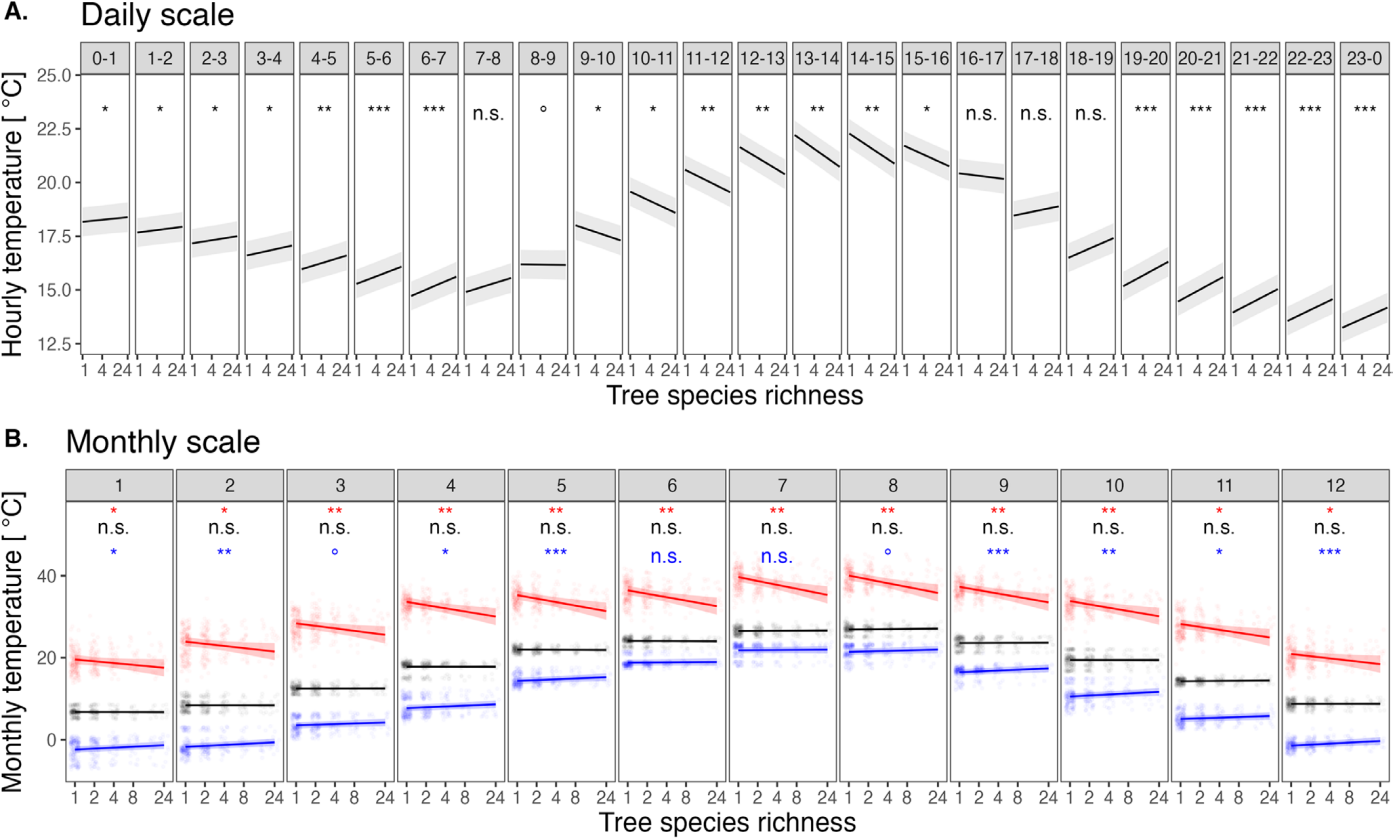
Tree species richness effects on microclimate temperature on (A) the daily and (B) the monthly scale. (A) Hourly modulation of microclimate temperatures by tree species richness (*n* = 63 plots and 4 million values; marginal *R* ^2^ = 0.16). (B) Monthly modulation of maximum (red), median (black) and minimum (blue) daily temperatures per month by tree species richness (*n* = 63 plots and 4476 values; marginal *R* ^2^ = 0.79, 0.98, and 0.96, for maximum, median and minimum temperature models). Lines show predictions of linear mixed‐effects models, and shaded bands indicate 95% confidence intervals. Data points in (B) are jittered to enhance visibility. Species richness was log‐transformed in all models. See Output S2 and S3 for complete model outputs. Significance levels: “n.s.”: Non‐significant, “°”: *p* < 0.1, “*”: *p* < 0.05, “**”: *p* < 0.01, and “***”: *p* < 0.001.

**FIGURE 2:**
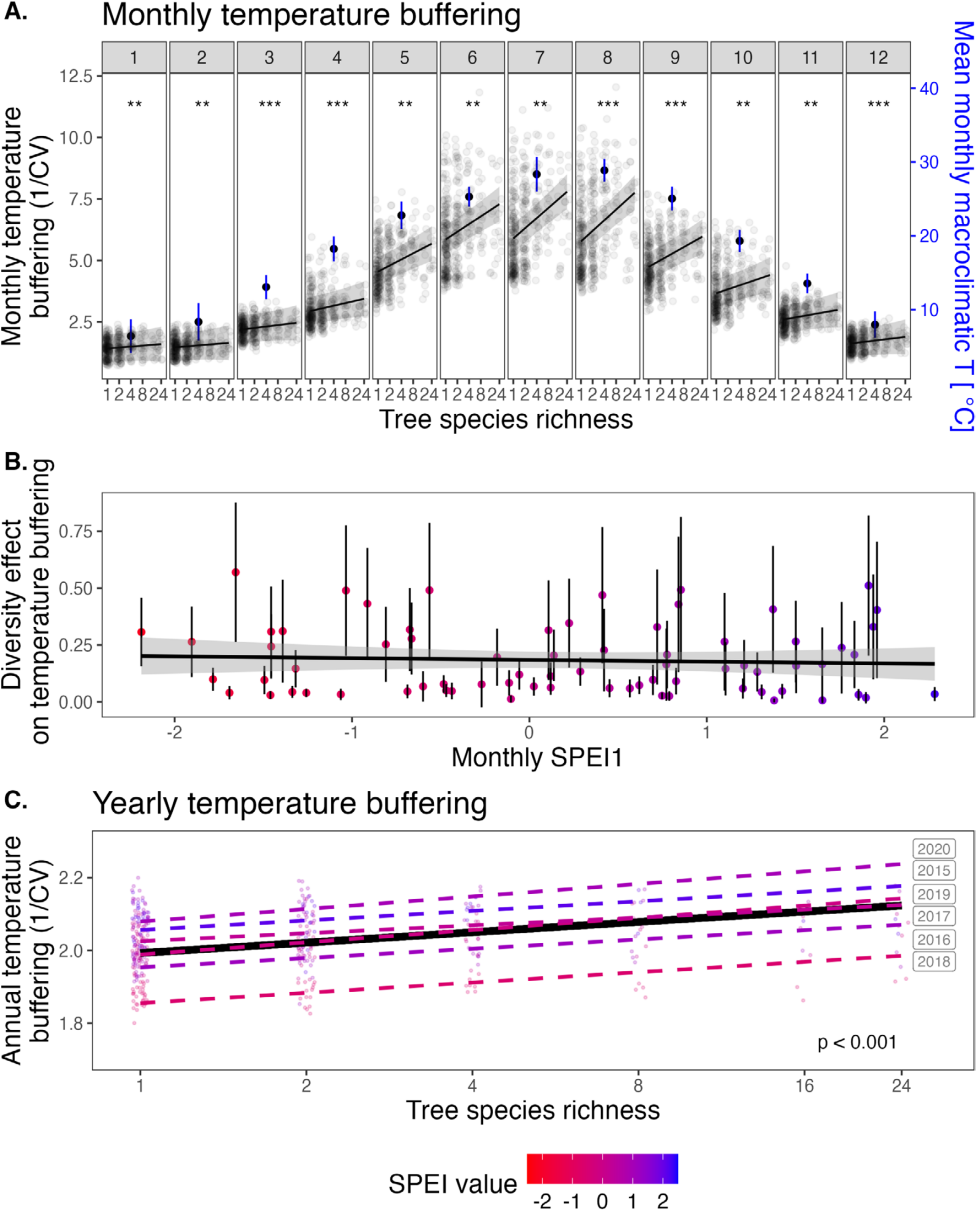
Tree species richness effects on temperature buffering on the monthly (A, B) and the annual scale (C). (A) Modulation of monthly microclimate temperature stability by tree species richness and month of the year (*n* = 63 plots and 4476 values; marginal *R* ^2^ = 0.77). (B) Diversity effects on monthly microclimate temperature stability according to drought severity. (C) Modulation of annual microclimate temperature stability by tree species richness and year (*n* = 63 plots and 375 values; marginal *R* ^2^ = 0.39). In all panels, the lines show predictions of linear mixed‐effects models. In (A), *p*‐values refer to the effects of species richness on monthly temperature buffering and solid black points show mean monthly macroclimate temperatures. In (B), points show monthly diversity effects, i.e. the slopes of the regressions between species richness and monthly microclimate temperature stability. In (C), the solid black line and *p*‐value refer to the effect of species richness across years, while the dashed coloured lines show model fits for each individual year. In (A, B) shaded bands and whiskers indicate 95% confidence intervals. In (A, C), data points are jittered to enhance visibility. In (B, C), points and lines are coloured according to their value with deeper red and blue indicating increasing and decreasing drought, respectively, based on monthly or annual values of the standardised precipitation evapotranspiration index (SPEI1, SPEI12). Species richness was log‐transformed in all models. See Output S4 and S5 for complete model outputs. Significance levels: “n.s.”: Non‐significant, “°”: *p* < 0.1, “*”: *p* < 0.05, “**”: *p* < 0.01, and “***”: *p* < 0.001.

To examine the mechanisms that may mediate tree species richness effects on temperature buffering, we used Structural Equation Models (SEMs). The hypothesis‐driven SEMs were informed by previous work, including from the herein‐examined experiment (see Method S1 for the conceptual model and the literature‐derived hypotheses). Specifically, we examined if canopy thickness, density, and structural diversity, captured by mean tree height, LAI, and SSCI, respectively, mediate tree species richness effects on temperature buffering. We accounted for potential correlations between these forest properties through including partial, non‐directional correlations between them. We controlled for monthly variations in macroclimate temperatures by dividing monthly temperature buffering values by monthly macroclimate temperature values. To capture potential temporal changes in the strength of the examined drivers, we fitted separated SEMs for each month. We explored how species richness affected temperature buffering via canopy thickness, density, and structural diversity in 2019 at site A, where we had measurements of all forest properties (except for LAI which was measured in 2014) and where temperature buffering was close to the mean response across years. To remain consistent with prior studies in our experiment, we fitted direct pathways between species richness and LAI and SSCI using the datasets and model structures from the original studies (Perles‐Garcia et al. 2021; Peng et al. 2017). Therefore, the tree species richness–forest properties models were fitted on larger plot sets (*n* = 32, 54, and 74 plots for mean tree height, LAI, and SSCI, respectively) than the forest properties–temperature buffering models fitted for the plots for which we had microclimate data and data on all examined forest properties (*n* = 27 plots, tree species richness ranging from 1–16 species). To prevent pseudo‐replication caused by measuring tree height, LAI, and SSCI on an annual basis, relationships between tree species richness and these forest properties were fitted using yearly datasets instead of monthly ones. In the tree species richness–LAI model, we included terms correcting for very large residual effects of few specific species in the examined tree communities following Schmid et al. (2017) as detailed in Peng et al. (2017). We assessed global model fit via Fisher's C statistic (*p* > 0.05) and the independence of variables with tests of direct separation (*p* < 0.05 for violation of independence) and posteriori, included partial, non‐directional correlations between non‐independent variables (Lefcheck 2016).

All data handling and statistical analyses were performed using the R statistical software version 4.1.3. Explanatory variables in the SEMs were centred and divided by one standard deviation using the ‘scale’ function. Tree species richness was log2‐transformed in all models. LMMs and individual SEM pathways were fit with the nlme package (Pinheiro et al. 2020) and SEMs with the piecewiseSEM package (Lefcheck 2016). Model assumptions (i.e., normality, independence and homogeneity of variance, and independence of explanatory variables) were tested with the ‘check_model’ function in the performance package (Lüdecke et al. 2021).

## 3 Results

On the daily scale, we found below‐canopy air temperatures to decrease with tree species richness during daytime, while they increased with species richness during the night (Figure 1A). Hence, the mode of tree species richness effects on microclimate temperature changed significantly with the diurnal course in macroclimate temperatures from positive (during cold night‐time hours) to negative (during hot day‐time hours; Figure 1A: interaction between species richness and hour significant at *p* < 0.001). Mitigating species richness effects on microclimate temperature were strongest at midday peak hours (mean temperature offsets of −2.5°C ± 0.2°C from noon to 3 pm) and positive effects were strongest around midnight (+0.4°C ± 0.04°C from 11 pm to 2 am) between stands with 1 and 24 tree species, respectively.

On the monthly scale, we examined maximum, minimum, and median daily microclimate temperatures across months (Figure 1B). We found that tree species richness significantly reduced maximum microclimate temperature across all months (January–December, *p*‐value range of slopes 0.002–0.033); this effect was strongest during summer (up to −4.4°C ± 0.6°C in 24‐species mixtures in July, *p* = 0.004) and during high macroclimate temperatures (Figures S8 and S9). Tree species richness also increased minimum microclimate temperatures in most months (September–May; *p*‐value range of slopes 0.001–0.053); this effect was strongest in winter (up to +1.1°C ± 0.2°C in 24‐species mixtures in December, *p* < 0.001), non‐significant during summer (June–August; *p* > 0.05), and strongest during low macroclimate temperatures (Figure S10). We found no significant effect of tree species richness on median monthly temperatures (Figure 1B; *p* > 0.5 for all months), i.e., species richness only affected temperature extremes. Hence, as hypothesised, tree species richness cooled hot and insulated against cold macroclimate temperatures, which contributed to a reduction of temperature extremes in species‐rich stands.

We quantified the temperature buffering capacity of a tree community on monthly and annual time scales. We found a consistently positive effect of tree species richness on monthly temperature buffering across the entire year (January–December; *p* ≤ 0.006 for all months), which was strongest in summer (June–August; Figure 2A) and during high macroclimate temperatures (Figure S11). Tree species richness also had significant positive effects on annual temperature buffering during all years examined (*p* < 0.001; Figure 2C). Effects of species richness on monthly and annual temperature buffering (slope of the species richness–temperature buffering relationship) did not change significantly with monthly and annual drought severity nor across years (Figure 2B,C), but the absolute temperature buffering capacity of the examined tree communities changed with macroclimatic conditions. Temperature buffering was significantly lowest during the driest year (i.e., 2018, the year with the lowest SPEI12 values, *p* < 0.001; Figure 2C; Output S6).

**FIGURE 3:**
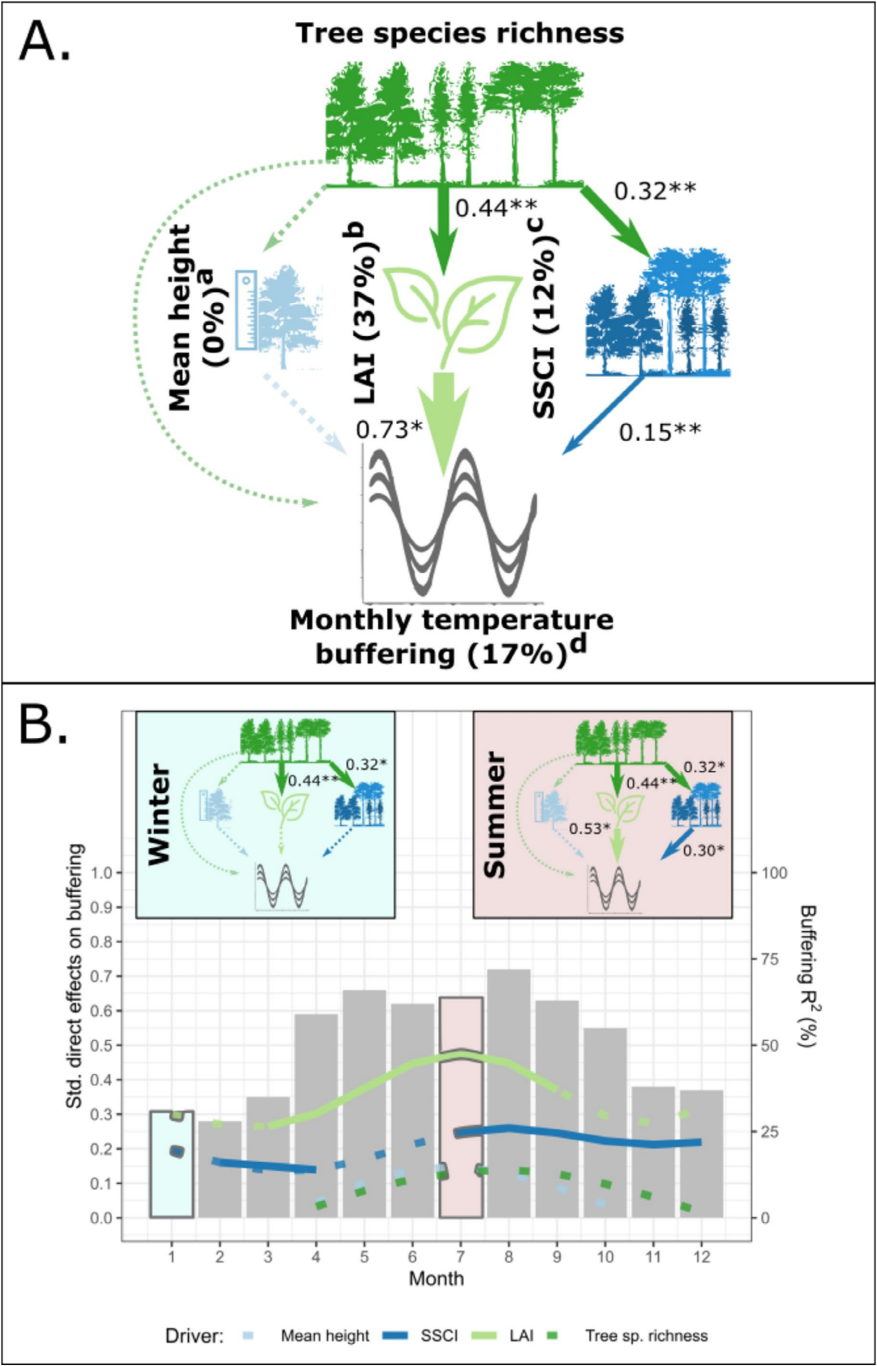
Structural Equation Models (SEMs) examining potential mediators of tree species richness effects on monthly temperature buffering. The SEMs test the direct effects of tree species richness and its indirect effects mediated by mean tree height, leaf area index (LAI), and stand structural complexity index (SSCI) on monthly temperature buffering while controlling for macroclimate temperatures. The SEM in (A) was fit to microclimate data of all months, and tree species richness effects on forest properties (i.e., mean tree height, LAI, and SSCI) were tested on the datasets built for this purpose (^a^32 plots, ^b^54 plots, ^c^74 plots, ^d^27 plots; see methods). All pathways were fit to data from site A measured in 2019 except for LAI, which was measured in 2014 (see Method S1 for details). Species richness and SSCI were log‐transformed in all models. Significant directional relationships between variables are shown as solid and nonsignificant relationships as dashed arrows. Significant standardised path coefficients are shown next to each path (*p < 0.05, **p < 0.01, and ***p < 0.001), and path width is scaled according to coefficient size. The explained variation of variables (marginal *R* ^2^) is given in %. The SEM fit the data well (Fisher's C = 19.1, df = 18, *p* = 0.38). In (B), the same SEM was fit separately for each month to explore temporal trends in the path coefficients. The SEMs in January and July exemplify pathways during winter and summer, respectively. For each month, coloured curves show standardised path coefficients (dashed if non‐significant) and bars show the variation in temperature buffering explained by the examined forest properties (marginal *R* ^2^); note that the marginal *R* ^2^ in (A) is lower than in the monthly models in (B) as it only captures the variation explained by fixed effects, which do not account for the strong variation in temperature buffering between months. See Output S7 and S8 for complete model outputs.

We used piecewise Structural Equation Models (SEMs; Figure 3) to examine potential mechanisms that may mediate the observed tree species richness effects on monthly temperature buffering (Figure 2). Once controlling for the effect of macroclimate, we found LAI to have the strongest positive effect on temperature buffering (Std. estimate = 0.73, *p* = 0.012), followed by SSCI (Std. estimate = 0.15, *p* = 0.002), while mean tree height had no significant effect on temperature buffering (*p* = 0.5, Figure 3A). Both LAI and SSCI significantly increased with increasing tree species richness (Std. estimate = 0.44, *p* = 0.007 and Std. estimate = 0.32, *p* = 0.003, respectively). Once accounting for these forest properties and their influence on temperature buffering, we found no remaining direct effect of tree species richness on temperature buffering (*p* = 0.3, Figure 3A). Using tree basal area measured in 2019 (another commonly used proxy for canopy‐ or stand density (Zhang et al. 2022; Gillerot et al. 2022)) instead of LAI resulted in similar pathways: tree species richness increased basal area (Std. estimate = 0.29, *p* = 0.049), which in turn enhanced temperature buffering (Std. estimate = 0.36, *p* = 0.011; Output S9). The influence of the different drivers changed over the annual course (Figure 3B): LAI was the strongest driver of temperature buffering during the growing season (March–September), while SSCI mostly affected temperature buffering before and after the growing season.

## 4 Discussion

In a large‐scale tree diversity experiment, we observed a consistent increase in forest temperature buffering across temporal scales with increasing tree species richness. Confirming H1, species‐rich tree communities cooled high and insulated against cold macroclimate temperatures better than species‐poor communities. This positive effect had a considerable magnitude with −4.4°C (± 0.6°C) and +1.1°C (± 0.2°C) in peak summer and winter for monocultures vs 24‐species mixtures, respectively. Confirming H2, temperature buffering was thus driven primarily by a reduction of maximum below‐canopy temperatures, with this effect being strongest during hot macroclimate conditions (during midday and summer). However, we acknowledge that the vertical temperature profiles in forests vary significantly (de Frenne et al. 2021) and that many ecosystem processes (e.g., soil respiration) are occurring not at one m above ground where we measured microclimate temperatures.

We expected species‐rich tree canopies to mainly cool hot temperatures by enhancing evapotranspiration and the reflection of short‐wave and emittance of long‐wave radiation (de Frenne et al. 2019; Geiger et al. 2009). Likewise, tree canopies may insulate against cold temperatures by retaining heat and long‐wave radiation, even though many more processes are likely involved (de Frenne et al. 2021). Consistent with our findings, stronger buffering of maximum relative to minimum temperatures predominates across the world's forests (de Frenne et al. 2021, 2019). Moreover, next to temperature extremes, droughts will likely threaten the world's forests during the 21st century (Hartmann et al. 2022; IPCC 2022). We found the lowest absolute temperature buffering in the driest year (2018) of our observation period (Figure 2C), likely due to reduced cooling potentials via evapotranspiration (as a result of the low atmospheric and soil moisture) (de Frenne et al. 2021; Greiser et al. 2024). However, the buffering role of tree species richness was maintained across a range of drought conditions (Figure 2), indicating that tree species richness provides insurance against climate extremes in the subtropical tree communities we studied. Still, it remains unclear if the buffering capacity of tree diversity can be maintained under intensifying climate change (IPCC 2022). In particular, severe tree mortality might affect our findings, which could be studied in the future by direct experimental manipulations of disturbance severity (Atkins et al. 2023).

There is ample evidence that forests buffer temperature extremes (de Frenne et al. 2019; Geiger et al. 2009; Kemppinen et al. 2024) and that species identities matter for temperature buffering (Ehbrecht et al. 2019; Zhang et al. 2022), but the role of tree diversity has largely remained hidden. The few former studies on the role of tree species composition for temperature buffering reported predominantly non‐significant effects of species richness (Ehbrecht et al. 2019; Gillerot et al. 2022; Donfack et al. 2021). Positive effects were rare and only found for specific mixtures (Zhang et al. 2022). It may be that idiosyncrasies of the investigated species prevented the detection of general patterns of species richness in earlier studies or that the level of species richness analysed was too low to detect significant effects. Our experimental design with a long tree species richness gradient ranging from 1 to 24 tree species and randomised extinction scenarios where each richness level was represented by different species compositions and each species occurred at each richness level (Bruelheide et al. 2014) allowed us to move beyond the effects of specific species compositions while controlling for environmental variation and species identity effects. Confirming this view, earlier studies in our experiment have demonstrated that most species and not only some particular species contributed to the observed diversity effects. For instance, complementarity and not selection effects drove the net positive tree diversity effects on stand volume (Huang et al. 2018).

Partially confirming H3, which we based on literature‐derived hypotheses (Table S2), we found positive tree species richness effects on temperature buffering to be mediated by enhanced canopy density and structural diversity but not by canopy thickness (Figure 3). The absence of a remaining direct tree species richness effect after accounting for these forest properties supports the use of the chosen proxies (LAI and SSCI) and suggests that we captured the dominant mechanisms driving temperature buffering. Still, monitoring other potential drivers, such as enhanced transpiration (Kunert et al. 2012; Wright and Francia 2024), will be relevant for comprehensively understanding species richness effects on temperature buffering. Canopy density and structural diversity were already shown to be enhanced by tree species richness in our experiment (Perles‐Garcia et al. 2021; Peng et al. 2017) and elsewhere (Schnabel et al. 2019; Barrufol et al. 2013; Ehbrecht et al. 2017). Likewise, canopy density (Gillerot et al. 2022; Zellweger et al. 2019; de Frenne et al. 2021) and structural diversity (Ehbrecht et al. 2019, 2017; Donfack et al. 2021) were reported to be significant drivers of forest temperature buffering. Moreover, and similar to our findings, structural diversity was more relevant than mere canopy height in this context (Ehbrecht et al. 2019). However, these studies did not elucidate the mechanistic links between species richness, canopy density, structural diversity, and temperature buffering, and it thus remained unclear what supported canopy density and structural diversity. Here, we provide experimental evidence that species richness bolsters temperature buffering by inducing changes in these forest properties. Furthermore, our study reveals that drivers of temperature buffering in forests exhibit temporal complementarity, with LAI being most relevant during the peak growing season and SSCI, which captures the structural diversity of canopy elements (stems and branches) during the leaf‐off period of the deciduous tree species, taking over outside the growing season.

The positive effect of tree diversity on temperature buffering we report represents, as also highlighted by recent advances in grassland research (Wright et al. 2021; Huang et al. 2024), a previously overlooked biodiversity‐ecosystem functioning (BEF) relationship, with potentially far‐reaching implications (Beugnon et al. 2024). In contrast to other mechanisms that cause positive BEF relationships in forests, such as biotic interactions between trees, negative density effects, or multitrophic interactions (Trogisch et al. 2021), which are all species‐specific, temperature buffering emerges from the community as a whole. The resulting lower temperature variation in species‐rich forests may safeguard ecosystem functions, particularly those that respond non‐linearly to temperature (Chapin III et al. 2011), against temperature maxima (and minima). This may be especially relevant for functions severely impeded beyond narrow threshold ranges of temperature, such as net photosynthesis rates (Hüve et al. 2011). Likewise, belowground functioning, including carbon sequestration, decomposition, and nutrient cycling (Gottschall et al. 2019; Seidelmann et al. 2016; Beugnon et al. 2023), may be enhanced by temperature buffering. As a result, trees in mixtures may grow (Schnabel et al. 2024) and regenerate better (Dobrowski et al. 2015) in ameliorated microclimates (Wright et al. 2017), which may, in turn, enhance temperature buffering via enhancing canopy density (Figure 3). Moreover, by reducing maximum temperatures (Figure 1), tree diversity–enhanced temperature buffering may impact forest biodiversity under global warming by reducing the thermophilization of below‐canopy communities (Zellweger et al. 2020; de Frenne et al. 2013). Finally, forest temperature buffering also alleviates heat stress for humans, and our findings indicate that tree species richness may amplify this effect far stronger than previously reported (Gillerot et al. 2022).

We suggest that preserving and planting diverse forests (Messier et al. 2021) is a promising approach to increase the temperature buffering function of forests, thereby protecting ecosystem functions and communities below the tree canopy against global warming. We compared the effects of increasing tree diversity on temperature buffering and the mediation of tree diversity effects by LAI and SSCI at constant planting density. Hence, at higher planting densities, mixtures would still outperform monocultures. Nonetheless, attempting to promote LAI and SSCI and, thereby, temperature buffering by planting monocultures with only a single or a few shade‐tolerant tree species may be theoretically possible. However, such species‐poor forests would have other well‐known limitations, such as a higher susceptibility to specialist pests and pathogens, droughts, and storms (Messier et al. 2021). In contrast, species‐rich forests are more likely to maintain their buffering capacity in the future (Zhang et al. 2022), given their higher stability under global change (Schnabel et al. 2021), while simultaneously providing a broader range of ecosystem services (Messier et al. 2021). Despite examining young planted forests (up to 11 years after establishment), we already detected a strong temperature buffering capacity. Still, our results only represent young forests, and it remains unclear how tree diversity affects temperature buffering as these tree communities grow and the examined forest properties change with stand development. We anticipate similar or stronger tree diversity effects on temperature buffering in older forests due to ample evidence for significant temperature buffering in mature forests (de Frenne et al. 2019) and increasing tree diversity effects on ecosystem functioning over time (Guerrero‐Ramírez et al. 2017). Overall, our findings thus highlight the benefits of diverse planted forests for large‐scale forest restoration initiatives and urban forests (Verheyen et al. 2024) that aim to reduce thermal stress in a warming world.

## Author Contributions

H.B., K.M., B.Y., W.H., P.A.N., G.v.O., B.S., and C.W. designed the experiment; F.S., R.B., and R.R. conceived the study; B.Y., H.B., F.S., R.B., X.L., A.F., M.D.P.G., G.J.A.H., W.H., M.K., N.C.C.I., P.A.N., G.v.O., and S.T. measured and/or compiled data; F.S., R.B., R.R., B.Y., N.E., Y.H., X.L., C.W., and H.B. developed and refined the analysis concept; R.B. analysed the data with support from F.S. and G.J.A.H.; F.S., R.B., R.R., B.Y., S.C., N.E., Y.H., C.W., and H.B. interpreted the data; R.B. created figures; F.S. wrote the manuscript with support from R.B.; F.S., R.B., B.Y., R.R., N.E., Y.H., C.W., S.C., A.F., M.D.P.G., G.J.A.H., W.H., M.K., X.L., N.C.C.I., P.A.N., G.v.O., B.S., S.T., M.W., K.M., and H.B. contributed substantially to revisions of drafts.

## Conflicts of Interest

The authors declare no conflicts of interest.

### Peer Review

The peer review history for this article is available at https://www.webofscience.com/api/gateway/wos/peer‐review/10.1111/ele.70096.

## Supporting information


Data S1.

## Data Availability

The datasets generated and analysed in the study are publicly available at https://zenodo.org/doi/10.5281/zenodo.13626945 and at the BEF‐China project repository, http://data.botanik.uni‐halle.de/bef‐china. All R scripts used for this study can be found in the same Zenodo repository, at https://zenodo.org/doi/10.5281/zenodo.13626945.
