# Supplementary material for: Tree Diversity Increases Forest Temperature Buffering via Enhancing Canopy Density and Structural Diversity
Source: Ecol Lett. 2025 Mar 21;28(3):e70096. doi: 10.1111/ele.70096 (PMC11928777; doi:10.1111/ele.70096)
Supplement: Supplementary file 1 — Data S1. [file ELE-28-0-s001.pdf]

Supplementary material

Schnabel, Beugnon, Yang et al.

Contents

Figure S1: BEF China location 3

Diversity treatment 4
Figure S2: plot distribution 4

Figure S3: Spatial cover 5

Temporal structure 6
Figure S4: Temporal cover 6
Figure S5: Temporal resolution 7
Table S1: Diversity levels 8

Method S1: Hypothesis driven SEM 9
Figure S6: SEM structure 9
Table S2: Relationships between the variables considered in the SEM. 10
Table S3: Forest properties. Potential variables describing tree canopy thickness, density and structural diversity available within the BEF-China experiment as well as their sample size, temporal extent and references for already published data. Selected variables for the SEMs highlighted in bold. 11

Data 13
Figure S7: Correlation between variables 13
Output S1: Variables summary 14

Monthly diversity effects *vs.* macroclimate 15
Figure S8: Maximum temperature 15
Figure S9: Median temperature 16
Figure S10: Minimum temperature 17
Figure S11: Temperature buffering 18

Statistical model outputs 19
Fig. 1 19
Output S2: Fig. 1.A. Daily model structure and outputs 19
Model structure 19
Assumption validation 19
Model summary 19
Output S3: Fig. 1.B. Monthly model structure and outputs 21
Maximum temperature 21
Model structure 21
Assumption validation 21
Summary 24
Median temperature 26
Model 26

Assumption validation . . . 26
Summary . . . 29
Minimum temperature . . . 31
Model . . . 31
Assumption validation . . . 31
Summary . . . 34
Fig. 2 . . . 36
Output S4: Fig. 2.A. Monthly buffering model structure and outputs . . . 36
Model . . . 36
Assumption validation . . . 36
Summary . . . 39
Output S5: Fig. 2.C. Yearly model structure and outputs . . . 41
Model . . . 41
Assumption validation . . . 41
Summary . . . 44
Output S6: SPEI model structure and outputs . . . 46
Model structure . . . 46
Model output . . . 46
Figure S12: comparison CRU and local measurements . . . 47

SEM models outputs **48**
Output S7: yearly scale SEM output . . . 48
Output S8: Monthly SEM model outputs . . . 50
Output S9: SEM model using tree basal area, model structure and outputs. . . . 51

Figure S1: BEF China location

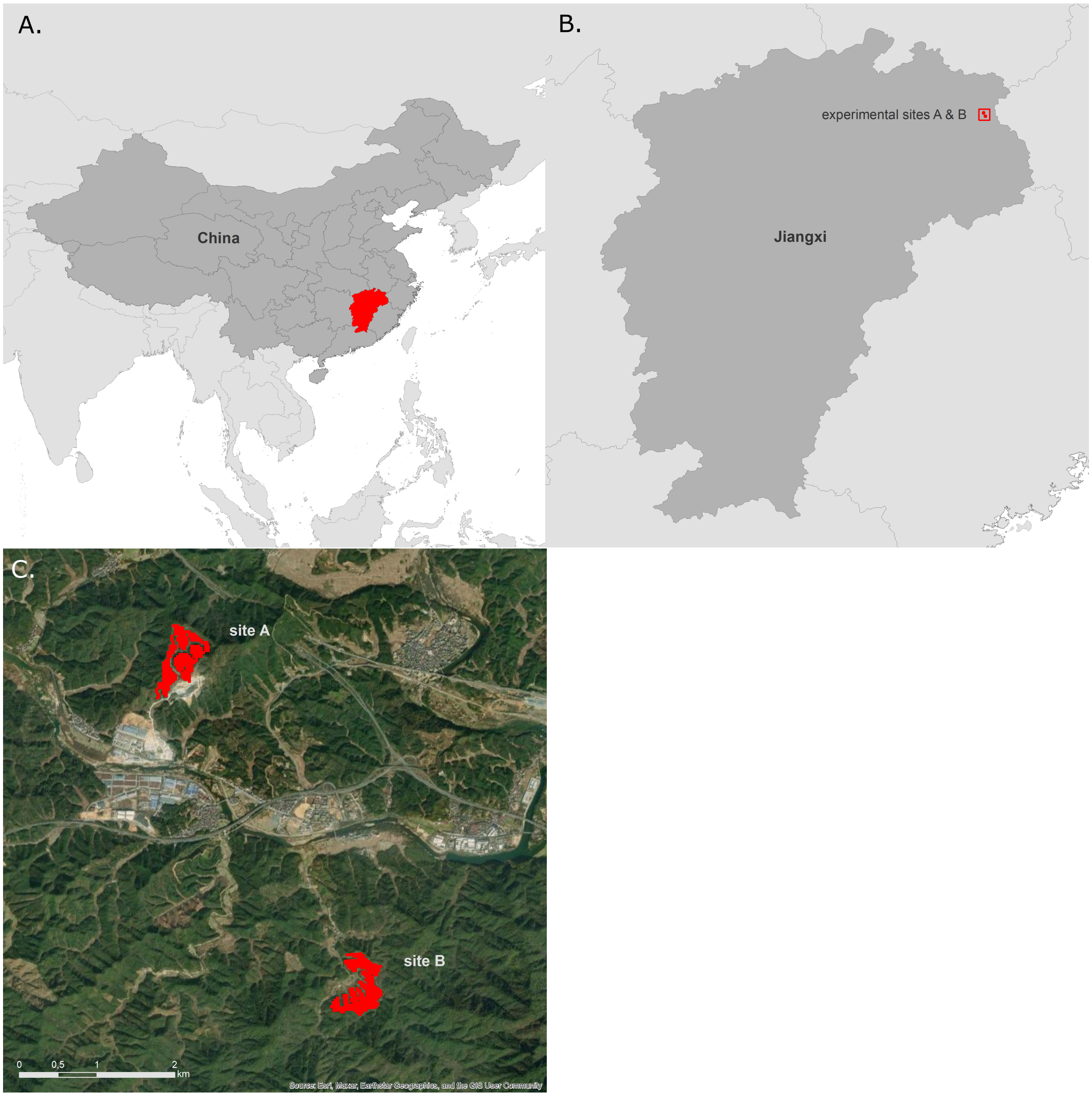

Figure S1: Geospatial location of the experiment, at country (A.), regional (B.) and landscape (C.) scales.

Diversity treatment

Figure S2: plot distribution

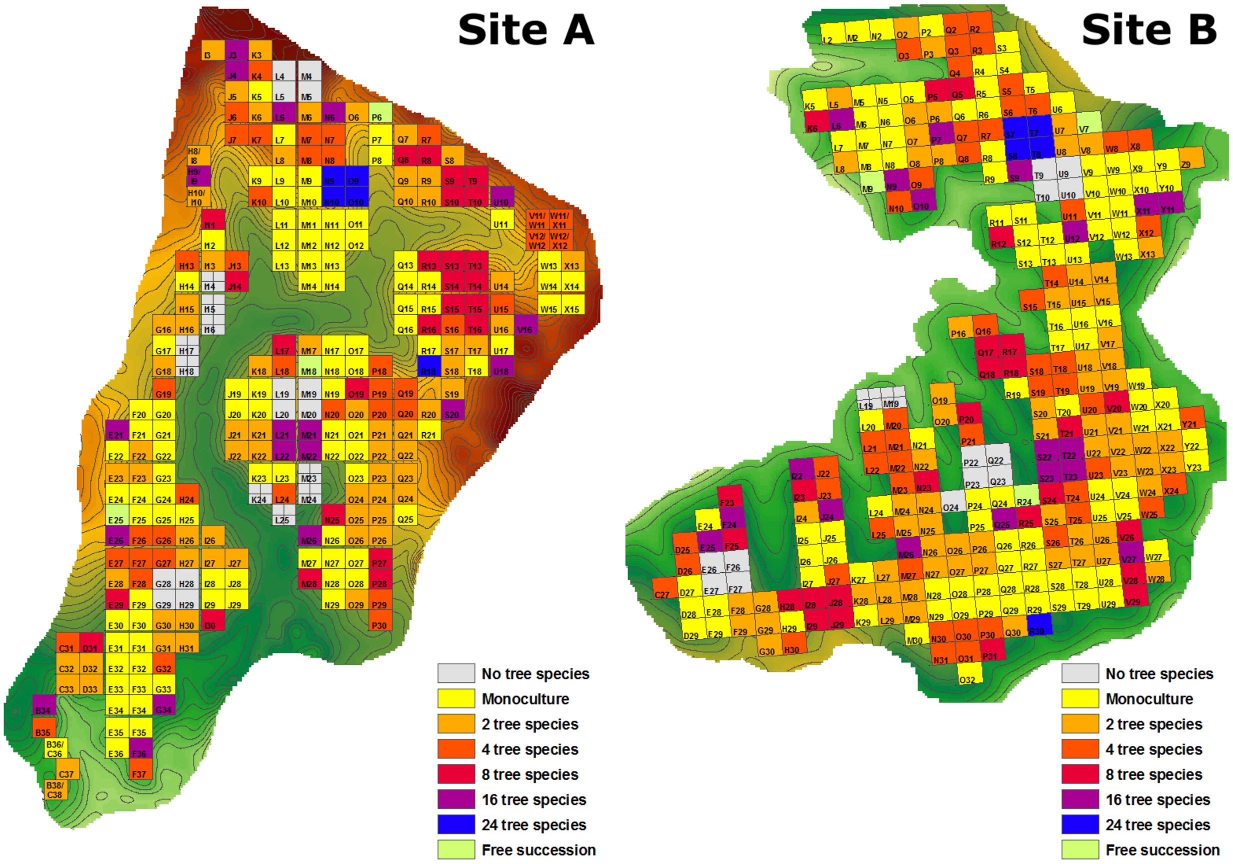

Figure S5: Position of plots covering a species richness gradient of 1 to 24 tree species within site A and B of the BEF-China experiment. We examined 32 VIP plots at site A and 31 VIP plots at site B covering the entire species richness gradient (the number of plots per richness level is shown in Table S1).

Figure S3: Spatial cover

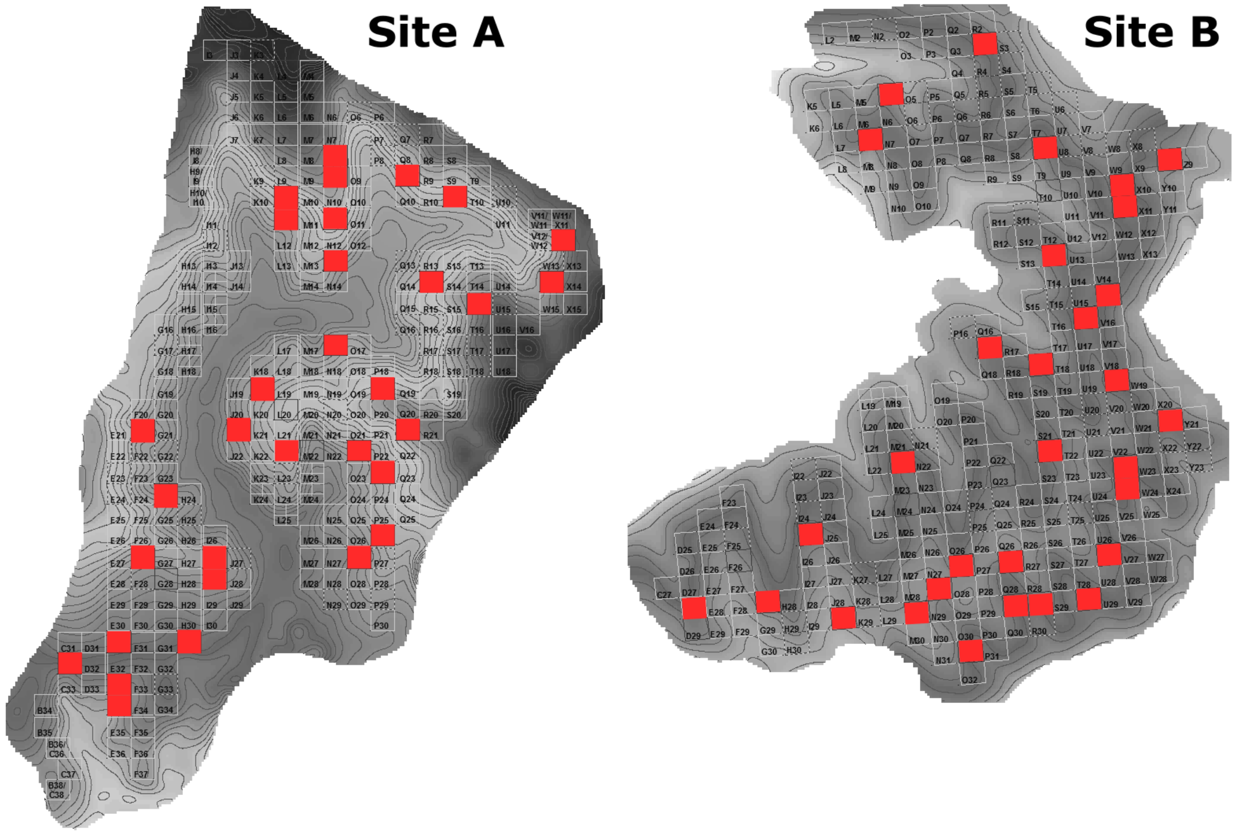

Figure S3: Position of the VIP plots within site A (n = 32) and B (n = 31) of the BEF-China experiment which are equipped with temperature loggers. Note, one plot at site B was excluded due to logger malfunction.

Temporal structure

Figure S4: Temporal cover

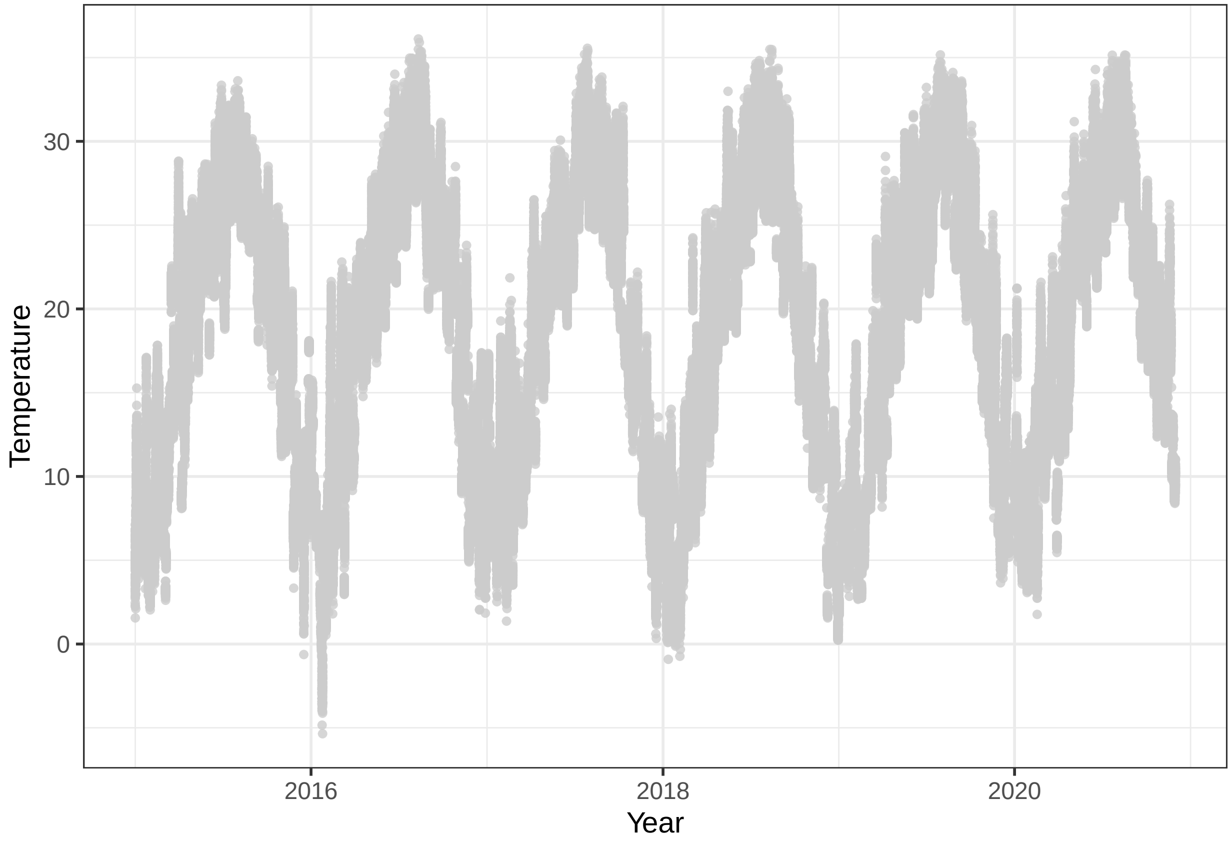

Figure S4: Time series of microclimate temperature measurements across years to illustrate the annual course of microclimate temperatures. Grey points shown hourly temperature measurements.

Figure S5: Temporal resolution

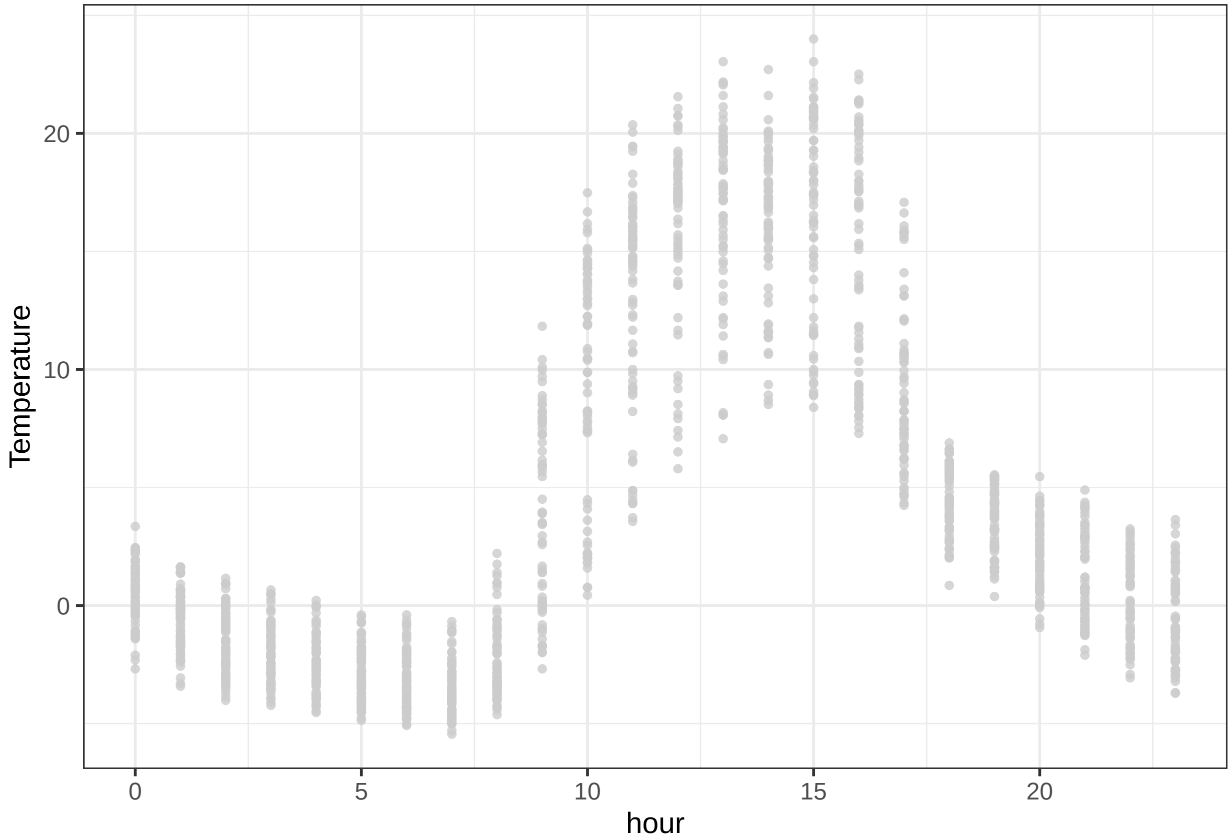

Figure S5: Resolution of the time series hourly measurement (here 01.01.2015) to illustrate the daily course of microclimate temperatures. Grey points shown hourly temperature measurements for all plots.

Table S1: Diversity levels

Tree species richness	Number of plots
1	32
2	16
4	7
8	4
16	2
24	2

Table S1: tree diversity experimental design

Method S1 Structural Equation Model (SEM) framework

We built a hypothesis-driven Structural Equation Model (SEM) framework to explain mechanisms behind observed tree species richness effects on temperature buffering (Fig. S5). This SEM framework was informed by prior knowledge of relationships between tree species richness and forest properties that in turn have been shown to affect temperature buffering (Table S2). However, the indirect effect of species richness via these forest properties on temperature buffering as well as their relative importance have been rarely assessed. We focussed on three potential pathways pertaining to tree canopy thickness, density and structural diversity (i.e. the variation of canopy elements) in three-dimensional space (Fig. S5). Based on literature-derived hypotheses (Table S2) we expected that tree species richness enhances canopy thickness, density and structural diversity (pathways 1, 3 and 5) and that these mediators in turn increase temperature buffering (pathways 2, 4 and 6). We additionally expected that species richness could potentially influence forest temperature buffering via processes not considered here (pathway 7).

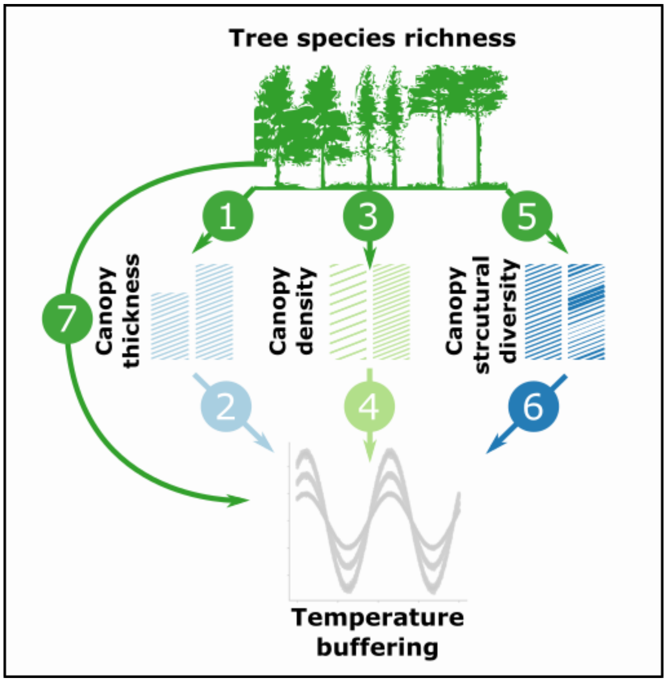

Figure S6 Hypothesis-driven SEM framework examining potential mediators of species richness effects on temperature buffering. Forest properties hypothesized to affect temperature buffering are show as sketches representing the thickness, density and structural diversity of aboveground canopy elements (e.g. leaves, branches or stems) at low (left) and high levels (right).

Table S2 Relationships between the variables considered in the SEM.

Forest property	Pathway	Hypothesized mechanism
Canopy thickness	1	Tree diversity can increase mean stand-level tree height (Vallet & Perot 2016).
	2	Mean stand-level tree height can increase temperature buffering (Ehbrecht *et al.* 2019; Gillerot *et al.* 2022).
Canopy density	3	Tree diversity can increase stand-level leaf area index (or basal area), through often-higher tree growth in mixtures (Huang *et al.* 2018; Schnabel *et al.* 2019; Peng *et al.* 2017; Jacob *et al.* 2010).
	4	Higher stand-level leaf area index (or basal area) can increase temperature buffering (Gillerot *et al.* 2022; Zhang *et al.* 2022; Frenne *et al.* 2021; Arx *et al.* 2013; Greiser *et al.* 2018).
Canopy structural diversity	5	Tree diversity can increase stand-level structural diversity, defined here as structural complexity in three-dimensional canopy space (SSCI index) (Juchheim *et al.* 2019; Perles-Garcia *et al.* 2021).
	6	Stand structural diversity can increase temperature buffering (Ehbrecht *et al.* 2017; Donfack *et al.* 2021; Ehbrecht *et al.* 2019).
Others	7	Species richness may also influence the temperature buffering of forests via other mechanisms not considered here. These mechanisms could include, but are not limited to, diversity-induced changes in branching patterns (Guillemot *et al.* 2020) and trait-driven effects that impact transpiration and water use such as leaf area, stomatal control traits, and rooting depth (Schnabel *et al.* 2024; Wright & Francia 2024).

To quantify canopy thickness, density and structural diversity, we assembled a range of variables from former studies and tree inventories in the BEF-China experiment. Overall, we could capture plot-level (i.e. stand-level) canopy thickness through data on mean tree height, mean crown length and mean crown base height, canopy density through the summed basal area and leaf area index (LAI) and structural diversity through the terrestrial laser scanning (TLS) derived effective number of layers (ENL) and structural complexity index (SSCI) (Ehbrecht *et al.* 2017; Ehbrecht *et al.* 2016) (Table S3). Tree basal diameter (5cm above ground level), height and crown base height were measured for the central 6 × 6 trees in each VIP plot to avoid edge effects and subsequently used to derive plot-level means for the inventory-based forest properties. Peng *et al.* (2017) detail the methods used to quantify LAI and Perles-Garcia *et al.* (2021) the methods used to measure ENL and SSCI. Of the potential variables (Table S3) we selected the ones with the highest relevance for

temperature buffering according to literature-derived hypothesis (Table S2), basically focussing on the ones which were most successfully used as predictors of temperature buffering in former studies. Specifically, we selected mean tree height, LAI and SSCI as proxies for canopy thickness, density and structural diversity, respectively. We selected LAI over basal area as we considered LAI to more specifically capture canopy density.

Table S3 Forest properties. Potential variables describing tree canopy thickness, density and structural diversity available within the BEF-China experiment as well as their sample size, temporal extent and references for already published data. Selected variables for the SEMs highlighted in bold.

**Forest property**	**Data**	**Number of plots**	**Temporal extent**	**Reference**
Canopy thickness	**Mean tree height**	**32**	**2019**	**Inventory; Unpublished data**
	Mean crown base height (i.e. height of lowest living branch)	32	2019	Inventory; Unpublished data
	Mean crown length (i.e. tree height - crown base height)	32	2019	Inventory; Unpublished data
Canopy density	**Leaf Area Index (LAI)**	**54**	**2014**	**Peng *et al.* (2017)**
	Summed basal area	32	2019	Inventory; Unpublished data
Canopy structural diversity	**Stand structural complexity index (SSCI)** based on TLS	**74**	**2019**	**Perles-Garcia *et al.* (2021)**
	Effective number of layers (ENL) based on TLS	74	2019	Perles-Garcia *et al.* (2021)

References

Arx, G. von, Graf Pannatier, E., Thimonier, A. & Rebetez, M. (2013). Microclimate in forests with varying leaf area index and soil moisture: potential implications for seedling establishment in a changing climate. *J. Ecol.*, 101, 1201–1213.

Donfack, L.S., Röll, A., Ellsäßer, F., Ehbrecht, M., Irawan, B. & Hölscher, D. *et al.* (2021). Microclimate and land surface temperature in a biodiversity enriched oil palm plantation. *For. Ecol. Manage.*, 497, 119480.

Ehbrecht, M., Schall, P., Ammer, C., Fischer, M. & Seidel, D. (2019). Effects of structural heterogeneity on the diurnal temperature range in temperate forest ecosystems. *For. Ecol. Manage.*, 432, 860–867.

Ehbrecht, M., Schall, P., Ammer, C. & Seidel, D. (2017). Quantifying stand structural complexity and its relationship with forest management, tree species diversity and microclimate. *Agric. For. Meteorol.*, 242, 1–9.

Ehbrecht, M., Schall, P., Juchheim, J., Ammer, C. & Seidel, D. (2016). Effective number of layers: A new measure for quantifying three-dimensional stand structure based on sampling with terrestrial LiDAR. *For. Ecol. Manage.*, 380, 212–223.

Frenne, P. de, Lenoir, J., Luoto, M., Scheffers, B.R., Zellweger, F. & Aalto, J. *et al.* (2021). Forest microclimates and climate change: Importance, drivers and future research agenda. *Glob. Chang. Biol.*, 27, 2279–2297.

Gillerot, L., Landuyt, D., Oh, R., Chow, W., Haluza, D. & Ponette, Q. *et al.* (2022). Forest structure and composition alleviate human thermal stress. *Glob. Chang. Biol.*

Greiser, C., Meineri, E., Luoto, M., Ehrlén, J. & Hylander, K. (2018). Monthly microclimate models in a managed boreal forest landscape. *Agric. For. Meteorol.*, 250-251, 147–158.

Guillemot, J., Kunz, M., Schnabel, F., Fichtner, A., Madsen, C.P. & Gebauer, T. *et al.* (2020). Neighbourhood-mediated shifts in tree biomass allocation drive overyielding in tropical species mixtures. *New Phytol.*, 228, 1256–1268.

Huang, Y., Chen, Y., Castro-Izaguirre, N., Baruffol, M., Brezzi, M. & Lang, A. *et al.* (2018). Impacts of species richness on productivity in a large-scale subtropical forest experiment. *Science*, 362, 80–83.

Jacob, M., Leuschner, C. & Thomas, F.M. (2010). Productivity of temperate broad-leaved forest stands differing in tree species diversity. *Ann. For. Sci.*, 67, 503.

Juchheim, J., Ehbrecht, M., Schall, P., Ammer, C. & Seidel, D. (2019). Effect of tree species mixing on stand structural complexity. *Forestry*, 93, 75–83.

Peng, S., Schmid, B., Haase, J. & Niklaus, P.A. (2017). Leaf area increases with species richness in young experimental stands of subtropical trees. *J. Plant Ecol.*, 10, 128–135.

Perles-Garcia, M.D., Kunz, M., Fichtner, A., Härdtle, W. & Oheimb, G. (2021). Tree species richness promotes an early increase of stand structural complexity in young subtropical plantations. *J. Appl. Ecol.*, 58, 2305–2314.

Schnabel, F., Barry, K.E., Eckhardt, S., Guillemot, J., Geilmann, H. & Kahl, A. *et al.* (2024). Neighbourhood species richness and drought-tolerance traits modulate tree growth and δ13C responses to drought. *Plant Biol.*, 26, 330–345.

Schnabel, F., Schwarz, J.A., Dănescu, A., Fichtner, A., Nock, C.A. & Bauhus, J. *et al.* (2019). Drivers of productivity and its temporal stability in a tropical tree diversity experiment. *Glob. Chang. Biol.*, 25, 4257-4272.

Vallet, P. & Perot, T. (2016). Tree diversity effect on dominant height in temperate forest. *For. Ecol. Manage.*, 381, 106–114.

Wright, A.J. & Francia, R.M. (2024). Plant traits, microclimate temperature and humidity: A research agenda for advancing nature-based solutions to a warming and drying climate. *J. Ecol.*, 112, 2462–2470.

Zhang, S., Landuyt, D., Verheyen, K. & Frenne, P. de (2022). Tree species mixing can amplify microclimate offsets in young forest plantations. *J. Appl. Ecol.*, 59, 1428–1439.

Data

Figure S7: Correlation between variables

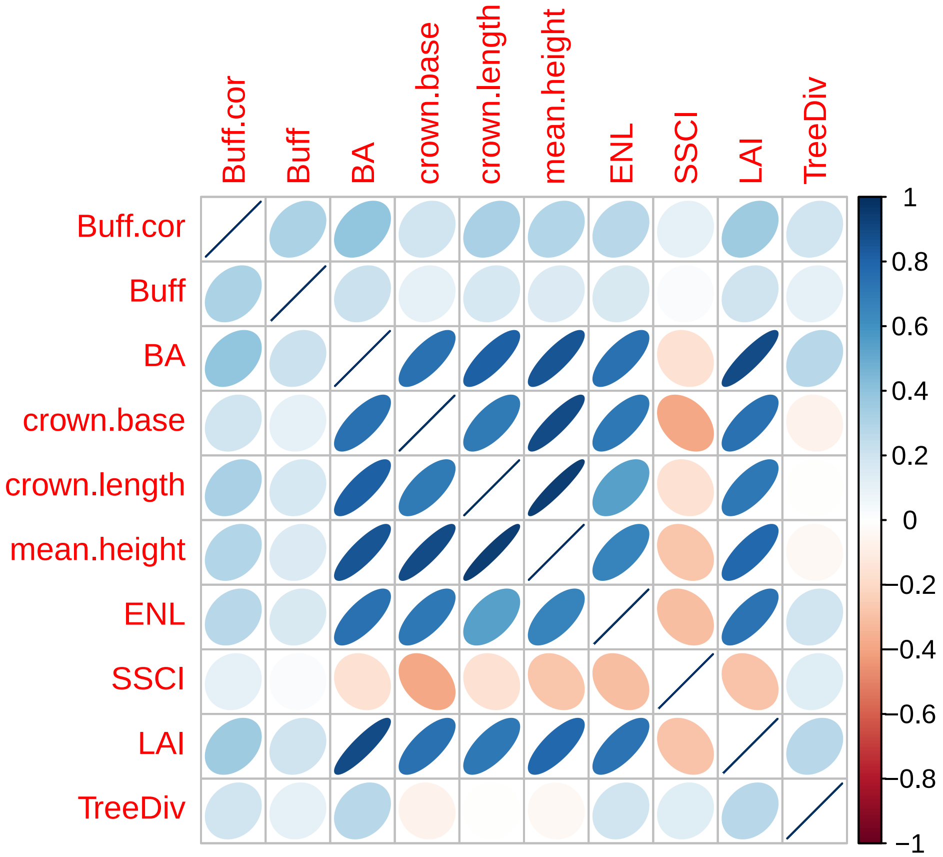

Correlation matrix between forest properties describing forest canopy thickness, density and structural diversity. Shown are Pearson's correlations between plot-level measurements of mean crown base height (crown.base), mean crown length (crown.length), and mean tree height (mean.height) as proxies for canopy thickness, basal area (BA) and leaf area index (LAI) as proxies for canopy density and effective number of layers (ENL) and structural complexity index (SSCI) as proxies for structural diversity. Correlations to monthly temperature buffering (Buff) and monthly temperature buffering controlled for macroclimate temperatures (Buff.cor) and tree species richness are included as well. Blue and red eclipses denote positive and negative correlations with thinner eclipses and darker colours showing stronger correlations; see the corrplot package in R for details.

Output S1: Variables summary

```
##      site               plot             crown.base      crown.length
##  Length:321         Length:321         Min.   :  0.00   Min.   :  0.0
##  Class :character   Class :character   1st Qu.: 54.31   1st Qu.:164.3
##  Mode  :character   Mode  :character   Median :104.67   Median :279.6
##                                        Mean   :124.15   Mean   :266.4
##                                        3rd Qu.:202.82   3rd Qu.:332.6
##                                        Max.   :360.28   Max.   :692.6
##   mean.height           ENL             SSCI              LAI
##  Min.   :-1.8872   Min.   :15.83   Min.   : 5.502   Min.   :-1.2005
##  1st Qu.:-0.8307   1st Qu.:23.39   1st Qu.:13.197   1st Qu.:-0.9216
##  Median :-0.1245   Median :31.59   Median :20.772   Median :-0.1470
##  Mean   : 0.0000   Mean   :34.60   Mean   :20.686   Mean   : 0.0000
##  3rd Qu.: 0.6964   3rd Qu.:44.37   3rd Qu.:25.870   3rd Qu.: 0.7762
##  Max.   : 2.6720   Max.   :70.10   Max.   :38.350   Max.   : 2.1339
```

Monthly diversity effects *vs.* macroclimate

Figure S8: Maximum temperature

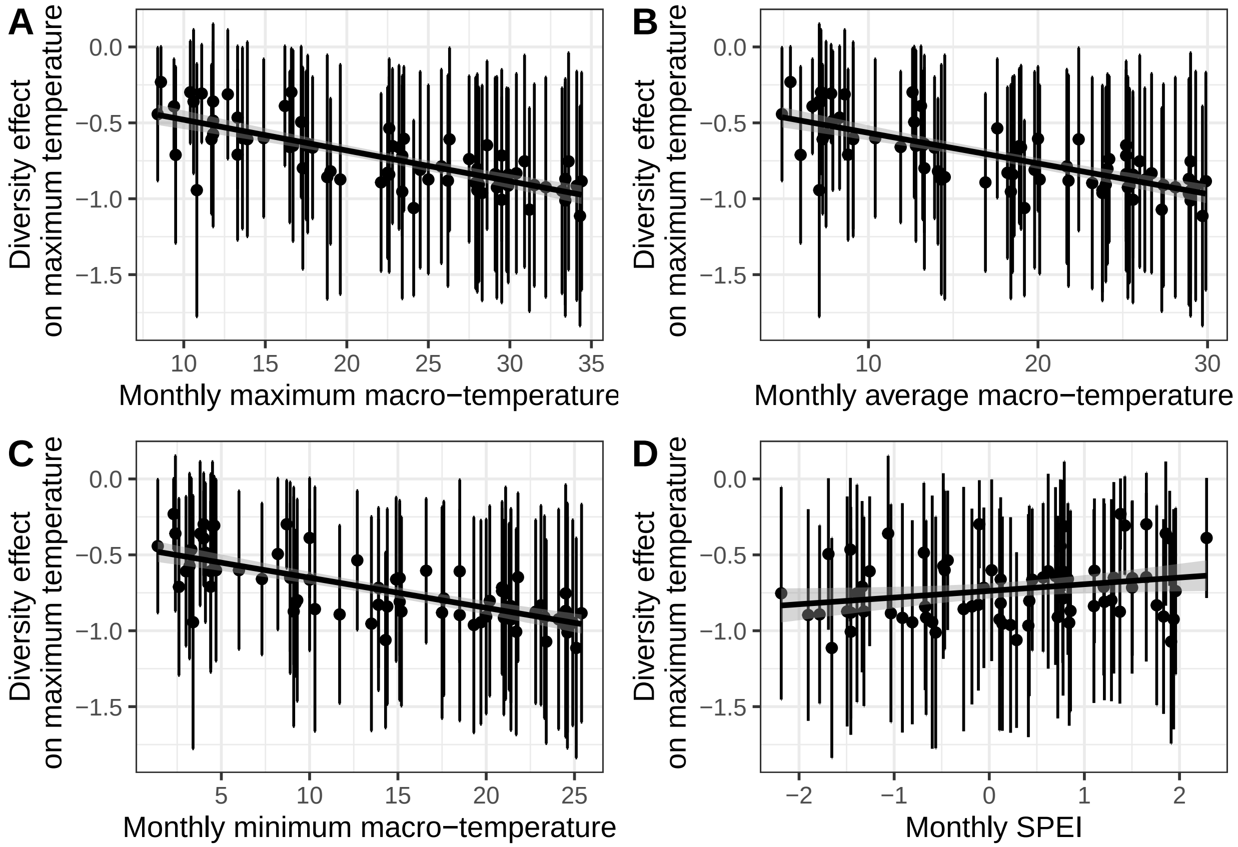

Figure S8: Macroclimate effects on the relationship between tree species richness and maximum microclimate temperature. Black points and error bars show diversity effects, i.e., slopes and respective standard errors, of the regression between maximum microclimate temperatures and tree species richness per month based on the model in Fig.1b (n = 63 for 12 months and 6 years). The change in these diversity effects with macroclimate conditions is examined for maximum (A), average (B) and minimum (C) monthly macroclimate temperatures and monthly values of the standardised precipitation evapotranspiration index (SPEI1; D). Trends are highlighted with a loess regression.

Figure S9: Median temperature

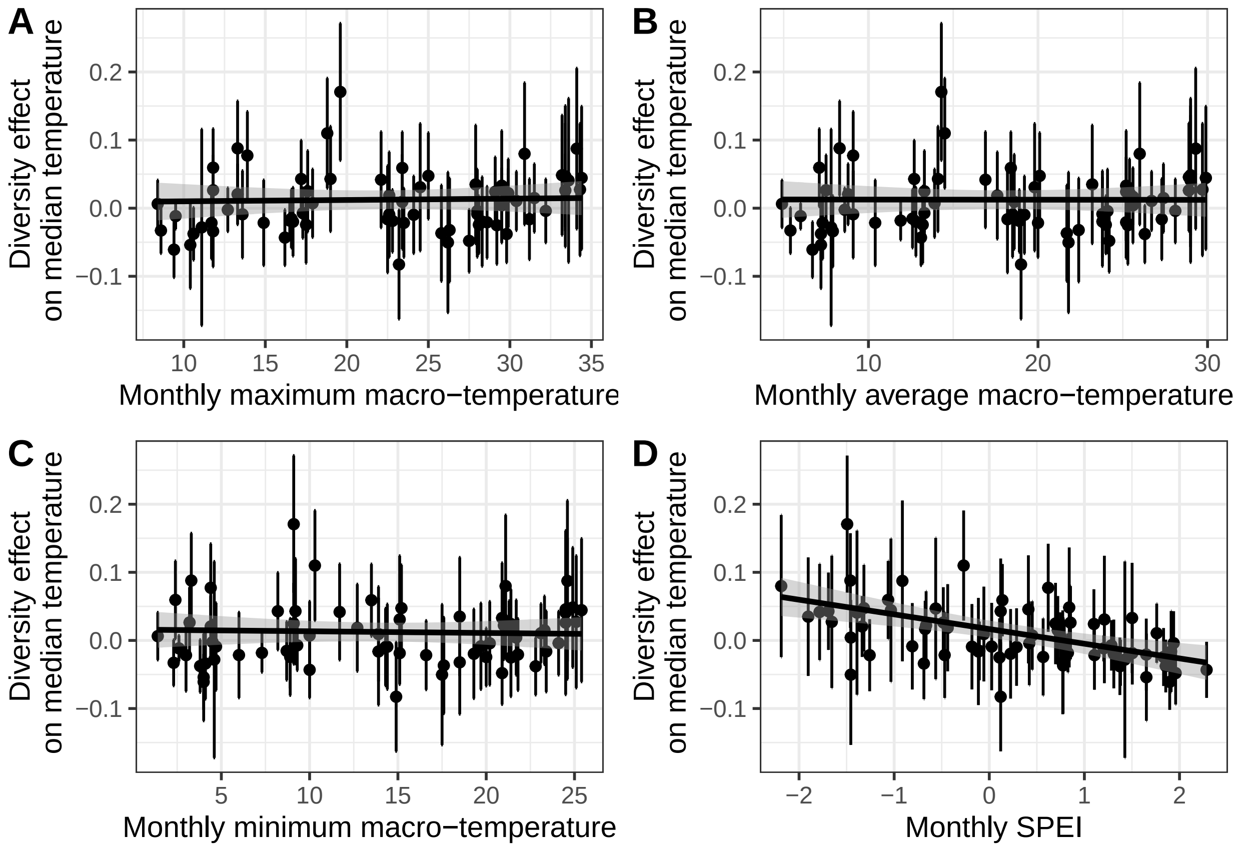

Figure S9: Macroclimate effects on the relationship between tree species richness and median microclimate temperature. Black points and error bars show diversity effects, i.e., slopes and respective standard errors, of the regression between median microclimate temperatures and tree species richness per month based on the model in Fig.1b (n = 63 for 12 months and 6 years). The change in these diversity effects with macroclimate conditions is examined for maximum (A), average (B) and minimum (C) monthly macroclimate temperatures and monthly values of the standardised precipitation evapotranspiration index (SPEI1; D). Trends are highlighted with a loess regression.

Figure S10: Minimum temperature

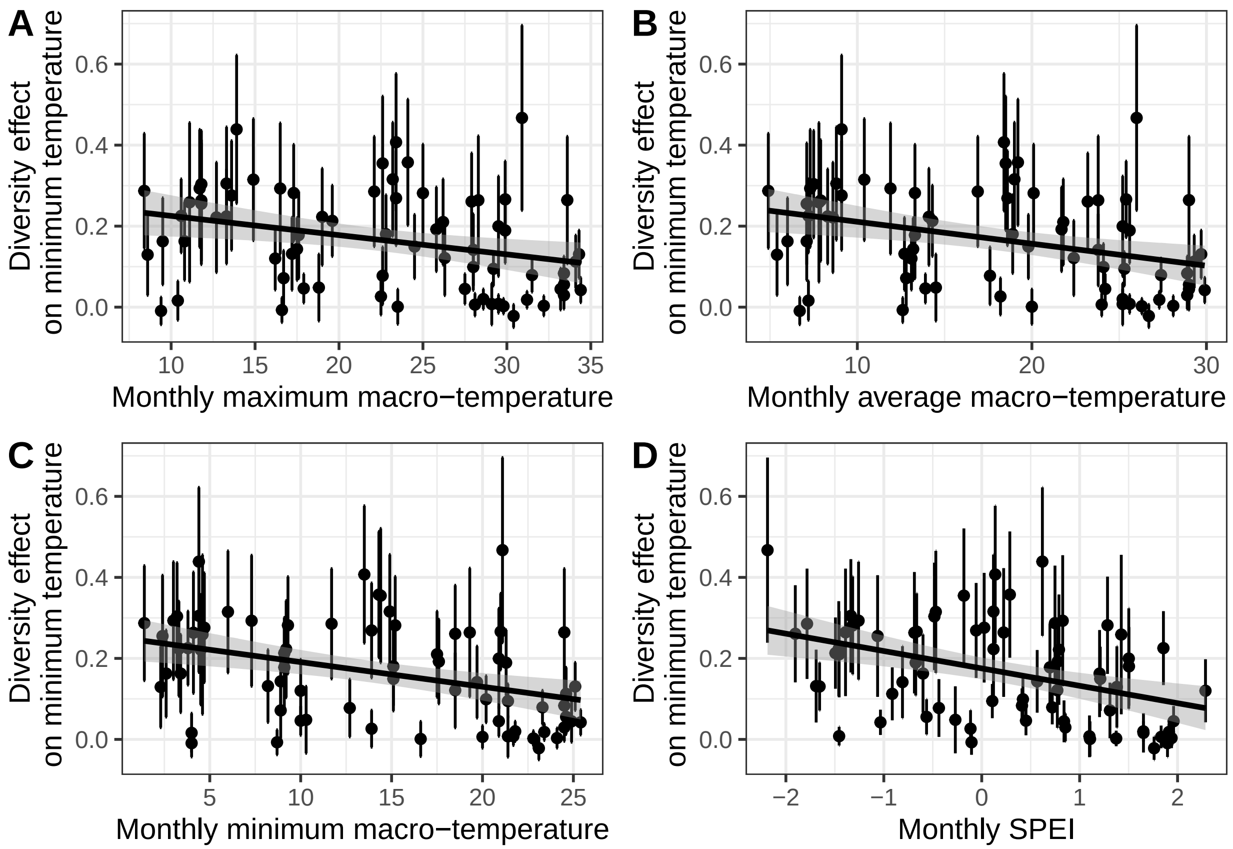

Figure S10: Macroclimate effects on the relationship between tree species richness and minimum microclimate temperature. Black points and error bars show diversity effects, i.e., slopes and respective standard errors, of the regression between minimum microclimate temperatures and tree species richness per month based on the model in Fig.1b (n = 63 for 12 months and 6 years). The change in these diversity effects with macroclimate conditions is examined for maximum (A), average (B) and minimum (C) monthly macroclimate temperatures and monthly values of the standardised precipitation evapotranspiration index (SPEI1; D). Trends are highlighted with a loess regression.

Figure S11: Temperature buffering

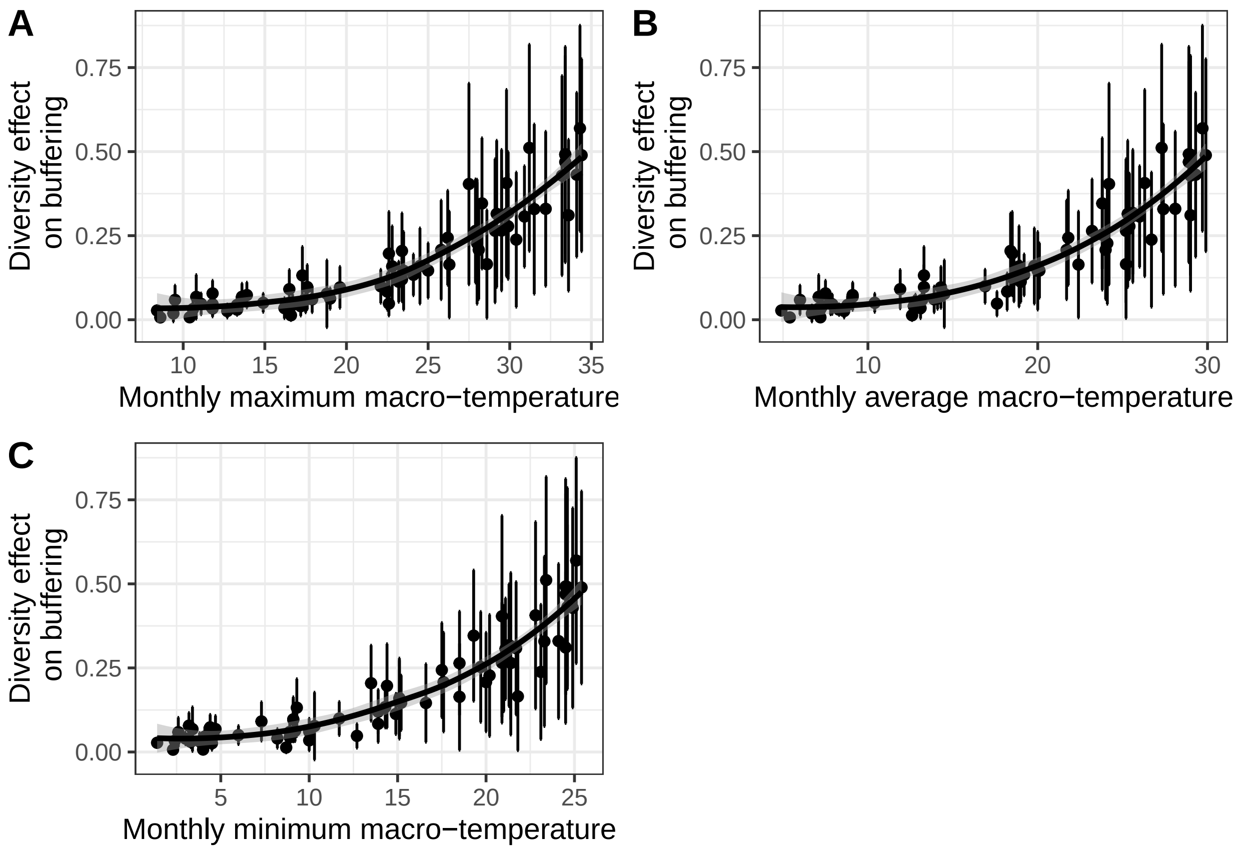

Figure S11: Macroclimate effects on the relationship between tree species richness and temperature buffering. Black points and error bars show diversity effects, i.e., slopes and respective standard errors, of the regression between temperature buffering and tree species richness per month based on the model in Fig.1b (n = 63 for 12 months and 6 years). The change in these diversity effects with macroclimate conditions is examined for maximum (A), average (B) and minimum (C) monthly macroclimate temperatures. Trends are highlighted with a loess regression.

Statistical model outputs

Fig. 1

Output S2: Fig. 1.A. Daily model structure and outputs

Model structure .

```
mod.daily =
  lme(T ~ log(TreeDiv) * hour.f,
      random = ~ 1|site/plot/date,
      data = data,
      correlation=corCAR1(),
      na.action=na.exclude)
```

.

Assumption validation .

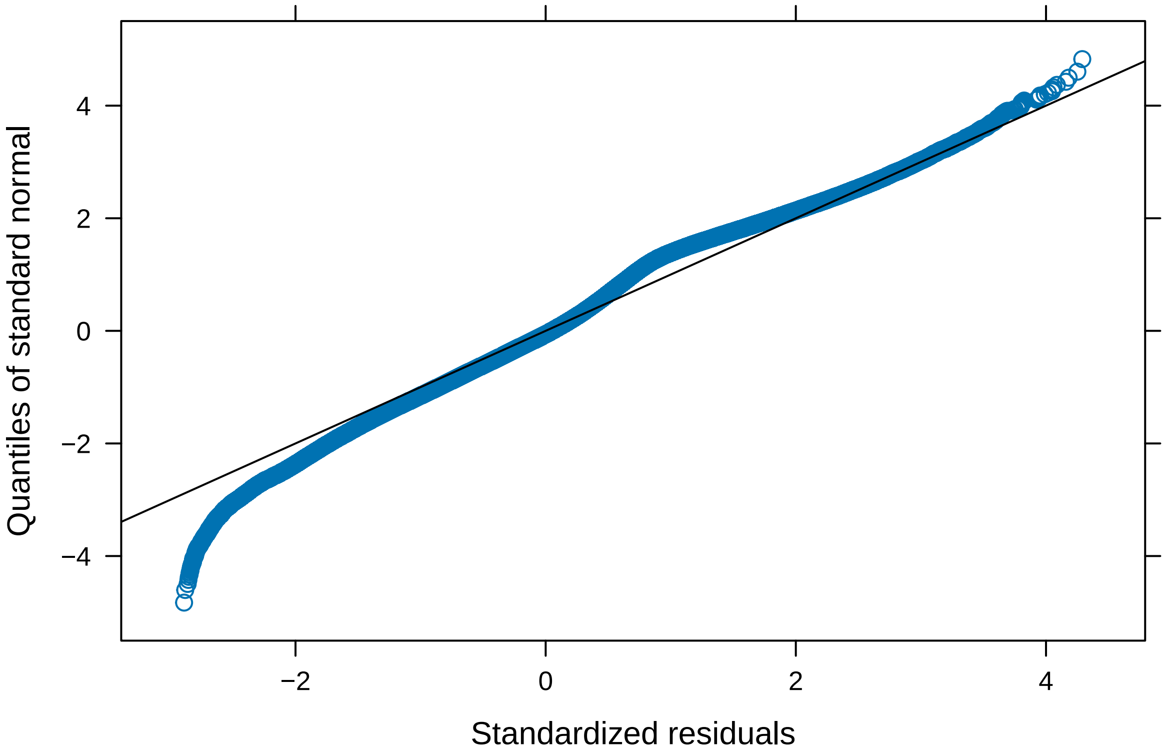

Model summary

```
  Response   family     link method  Marginal Conditional
1        T gaussian identity   none 0.1643892   0.6865668

                             Value  Std.Error     DF     t-value       p-value
(Intercept)           16.461109580 0.29598888 699945  55.6139464  0.000000e+00
log(TreeDiv)           0.233754431 0.06120162     60   3.8194160  3.196367e-04
hour.f1               -0.353838995 0.01010693 699945 -35.0095347 2.755591e-268
hour.f2               -0.671057326 0.01406768 699945 -47.7020751  0.000000e+00
```

hour.f3	-0.954797225	0.01695454	699945	-56.3151133	0.000000e+00
hour.f4	-1.192269321	0.01926166	699945	-61.8985853	0.000000e+00
hour.f5	-1.408960925	0.02118343	699945	-66.5123944	0.000000e+00
hour.f6	-1.474335370	0.02282088	699945	-64.6046562	0.000000e+00
hour.f7	-0.770207530	0.02423462	699945	-31.7812868	1.690983e-221
hour.f8	1.113968491	0.02546441	699945	43.7461010	0.000000e+00
hour.f9	3.622253242	0.02653790	699945	136.4936100	0.000000e+00
hour.f10	5.915129984	0.02747515	699945	215.2901470	0.000000e+00
hour.f11	7.497673735	0.02829174	699945	265.0128400	0.000000e+00
hour.f12	8.452801951	0.02899930	699945	291.4829708	0.000000e+00
hour.f13	8.865540613	0.02960540	699945	299.4568608	0.000000e+00
hour.f14	8.861762002	0.03011680	699945	294.2464918	0.000000e+00
hour.f15	8.260187516	0.03053863	699945	270.4832452	0.000000e+00
hour.f16	6.959912636	0.03087471	699945	225.4243568	0.000000e+00
hour.f17	4.875093606	0.03112774	699945	156.6157099	0.000000e+00
hour.f18	2.597058096	0.03129941	699945	82.9746697	0.000000e+00
hour.f19	1.050187680	0.03139049	699945	33.4556025	3.338612e-245
hour.f20	0.198676082	0.03140088	699945	6.3270860	2.499842e-10
hour.f21	-0.439222499	0.03133058	699945	-14.0189698	1.210087e-44
hour.f22	-0.953018736	0.03117739	699945	-30.5676270	4.506214e-205
hour.f23	-1.372137166	0.03093834	699945	-44.3507085	0.000000e+00
log(TreeDiv):hour.f1	-0.007143661	0.00931026	699945	-0.7672892	4.429099e-01
log(TreeDiv):hour.f2	-0.014790095	0.01295845	699945	-1.1413471	2.537259e-01
log(TreeDiv):hour.f3	-0.025269709	0.01561726	699945	-1.6180628	1.056495e-01
log(TreeDiv):hour.f4	-0.038393480	0.01774189	699945	-2.1640013	3.046459e-02
log(TreeDiv):hour.f5	-0.047875770	0.01951145	699945	-2.4537267	1.413868e-02
log(TreeDiv):hour.f6	-0.079430902	0.02101899	699945	-3.7790059	1.574687e-04
log(TreeDiv):hour.f7	-0.238110705	0.02232036	699945	-10.6678696	1.445663e-26
log(TreeDiv):hour.f8	-0.557472710	0.02345217	699945	-23.7706192	7.540890e-125
log(TreeDiv):hour.f9	-0.839211416	0.02443991	699945	-34.3377388	3.528071e-258
log(TreeDiv):hour.f10	-0.960315656	0.02530206	699945	-37.9540523	6.941835e-315
log(TreeDiv):hour.f11	-0.975222227	0.02605291	699945	-37.4323695	2.344701e-306
log(TreeDiv):hour.f12	-1.017111978	0.02670322	699945	-38.0894809	4.057498e-317
log(TreeDiv):hour.f13	-1.049806289	0.02725995	699945	-38.5109354	0.000000e+00
log(TreeDiv):hour.f14	-0.995745313	0.02772931	699945	-35.9094936	3.933989e-282
log(TreeDiv):hour.f15	-0.829690508	0.02811600	699945	-29.5095448	2.848326e-191
log(TreeDiv):hour.f16	-0.617270705	0.02842355	699945	-21.7168736	1.539264e-104
log(TreeDiv):hour.f17	-0.351687831	0.02865441	699945	-12.2734287	1.268479e-34
log(TreeDiv):hour.f18	-0.162450362	0.02881011	699945	-5.6386586	1.714456e-08
log(TreeDiv):hour.f19	-0.064458253	0.02889134	699945	-2.2310578	2.567762e-02
log(TreeDiv):hour.f20	-0.043365917	0.02889799	699945	-1.5006552	1.334452e-01
log(TreeDiv):hour.f21	-0.040297824	0.02883002	699945	-1.3977731	1.621817e-01
log(TreeDiv):hour.f22	-0.047151700	0.02868541	699945	-1.6437519	1.002279e-01
log(TreeDiv):hour.f23	-0.054777525	0.02846121	699945	-1.9246380	5.427507e-02

	numDF	denDF	F-value	p-value
(Intercept)	1	699945	3322.374	<.0001
log(TreeDiv)	1	60	2.193	0.1439
hour.f	23	699945	20411.297	<.0001
log(TreeDiv):hour.f	23	699945	206.598	<.0001

Output S3: Fig. 1.B. Monthly model structure and outputs

Maximum temperature

```
mod.monthly.Tmax =
  lme(T.max ~ log(TreeDiv, base = 2) * month.f,
      random = ~ 1|site/plot/year,
      data = data,
      correlation=corCAR1(),
      na.action=na.exclude)
```

Model structure

Assumption validation

```
$NCV
```

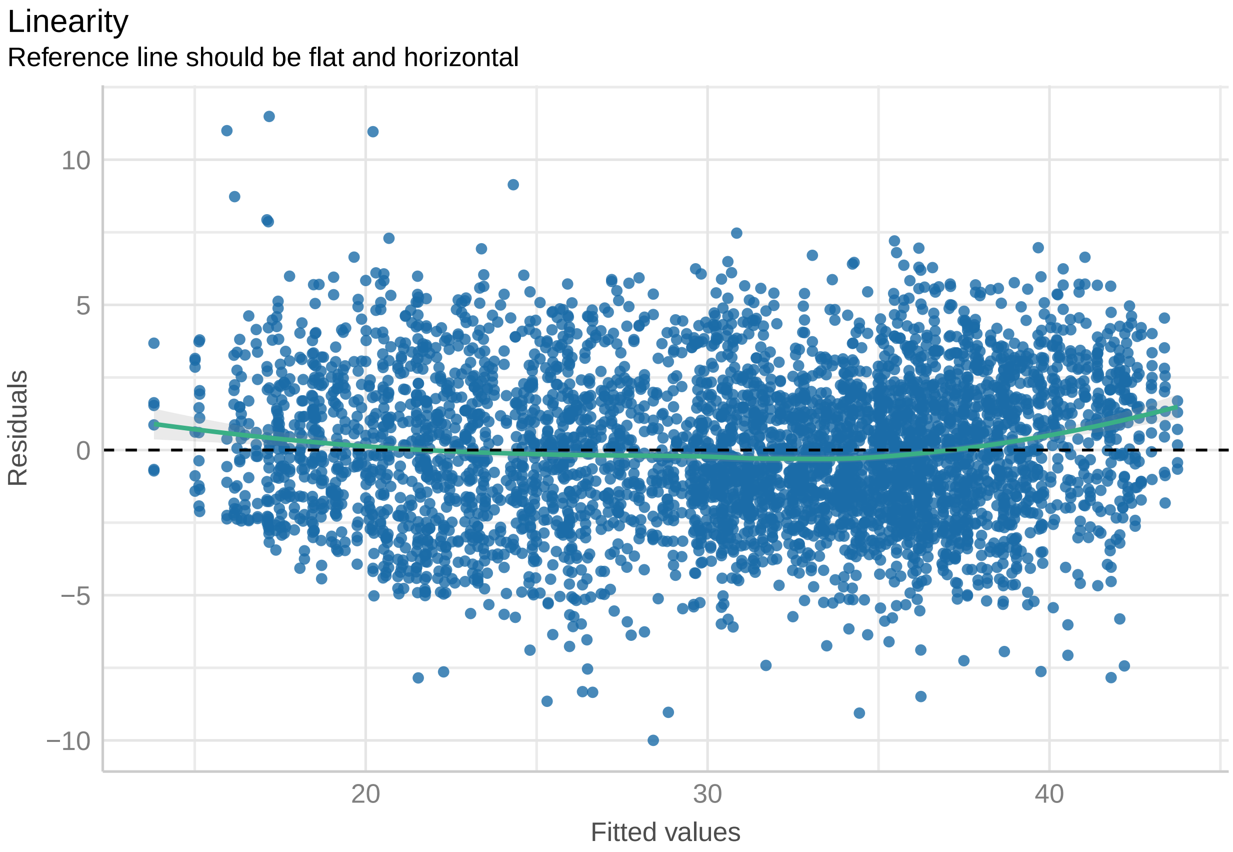


```
$VIF
```

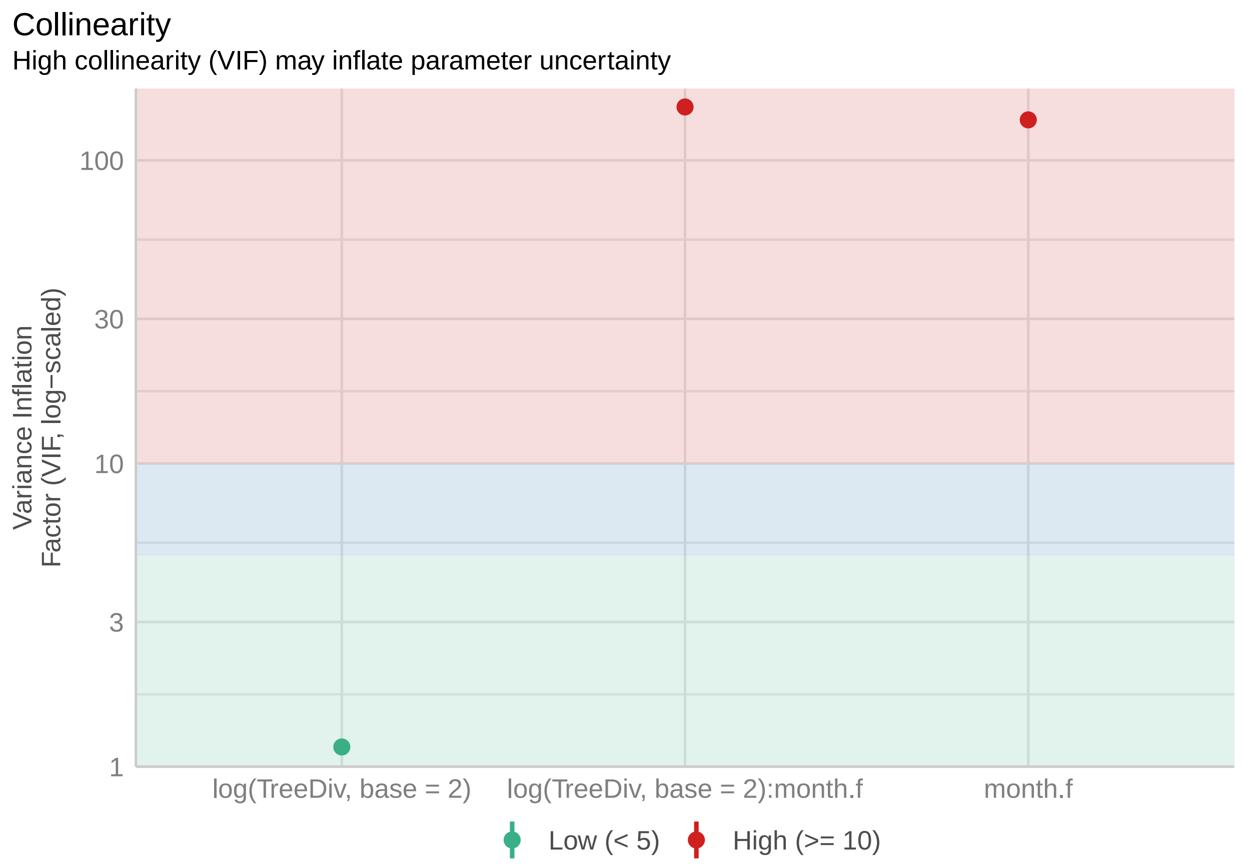
Collinearity
High collinearity (VIF) may inflate parameter uncertainty
Variance Inflation
Factor (VIF, log−scaled)
100
30
10
3
1
log(TreeDiv, base = 2)
log(TreeDiv, base = 2):month.f
month.f
Low (< 5)
High (>= 10)

$QQ

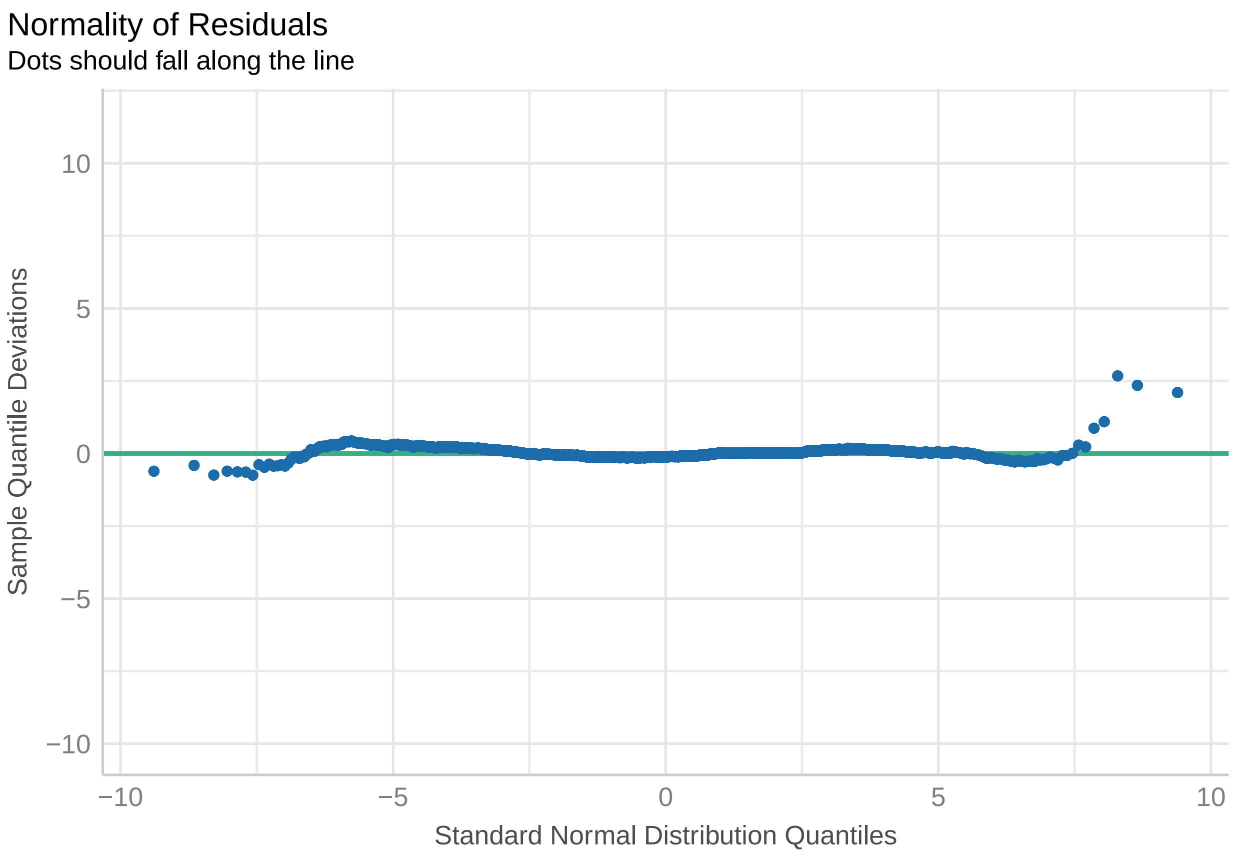
Normality of Residuals
Dots should fall along the line
Sample Quantile Deviations
10
5
0
−5
−10
−10
−5
0
5
10
Standard Normal Distribution Quantiles

```
$NORM
```

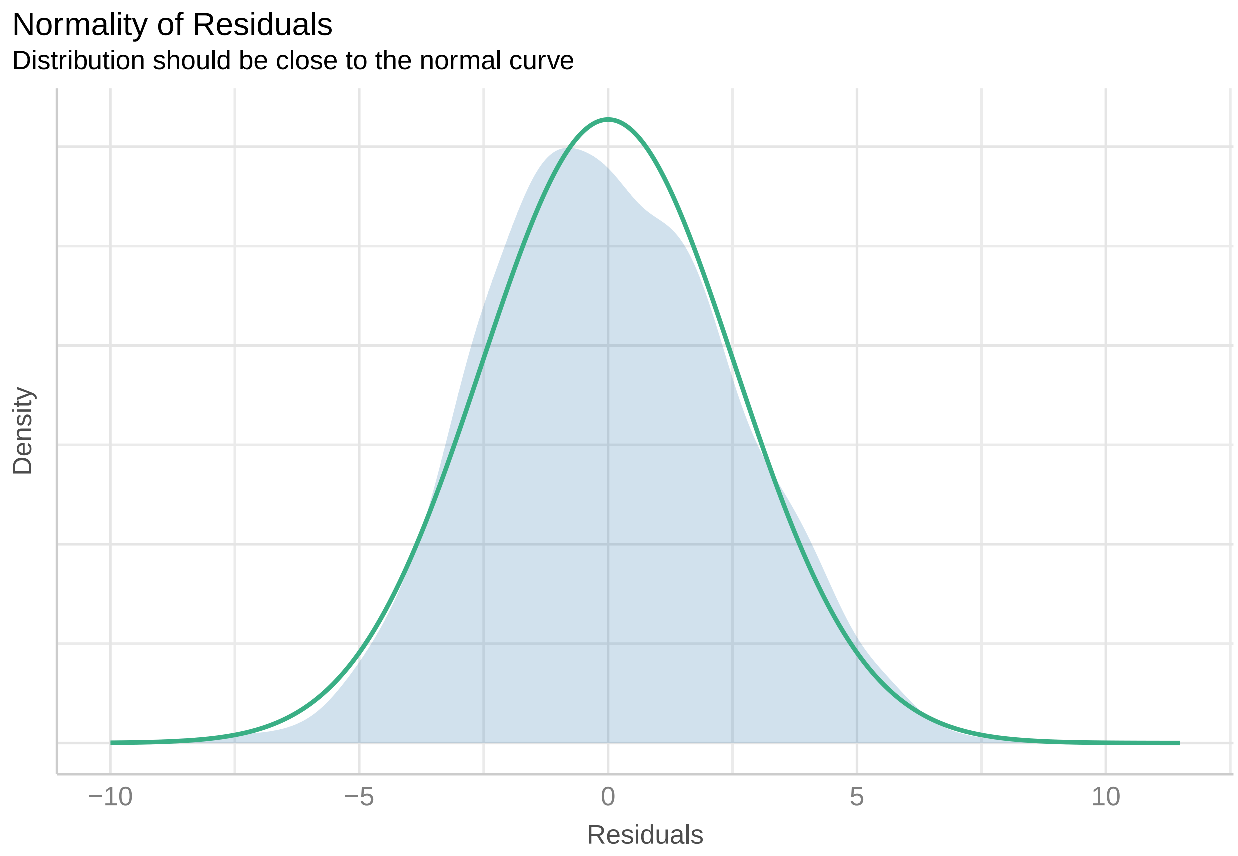

Summary

	Response	family	link	method	Marginal	Conditional
1	T.max	gaussian	identity	none	0.7852561	0.882336

	Value	Std.Error	DF	t-value
(Intercept)	19.57766903	0.5817134	4079	33.6551784
log(TreeDiv, base = 2)	-0.43824335	0.2537465	60	-1.7270913
month.f2	4.36477422	0.1659925	4079	26.2950108
month.f3	8.80419707	0.2042676	4079	43.1012849
month.f4	14.05269258	0.2211456	4079	63.5449885
month.f5	15.69114115	0.2294448	4079	68.3874360
month.f6	16.86451156	0.2336064	4079	72.1919860
month.f7	20.09719459	0.2357222	4079	85.2579557
month.f8	20.45908892	0.2368049	4079	86.3964074
month.f9	17.71960854	0.2374308	4079	74.6306136
month.f10	14.24044749	0.2379099	4079	59.8564626
month.f11	8.65274119	0.2380927	4079	36.3419023
month.f12	1.33768140	0.2381159	4079	5.6177743
log(TreeDiv, base = 2):month.f2	-0.09123946	0.1054842	4079	-0.8649588
log(TreeDiv, base = 2):month.f3	-0.16797419	0.1298339	4079	-1.2937617
log(TreeDiv, base = 2):month.f4	-0.34357585	0.1406828	4079	-2.4422018
log(TreeDiv, base = 2):month.f5	-0.40972801	0.1459787	4079	-2.8067655
log(TreeDiv, base = 2):month.f6	-0.40007604	0.1486341	4079	-2.6916837
log(TreeDiv, base = 2):month.f7	-0.50955502	0.1499841	4079	-3.3973926
log(TreeDiv, base = 2):month.f8	-0.48352234	0.1506751	4079	-3.2090392
log(TreeDiv, base = 2):month.f9	-0.38887356	0.1510301	4079	-2.5748082

```
log(TreeDiv, base = 2):month.f10 -0.36869841 0.1512533 4079 -2.4376217
log(TreeDiv, base = 2):month.f11 -0.27510810 0.1513581 4079 -1.8175971
log(TreeDiv, base = 2):month.f12 -0.09992012 0.1514093 4079 -0.6599338
                                      p-value
(Intercept)                      2.384471e-219
log(TreeDiv, base = 2)            8.929651e-02
month.f2                         6.638462e-141
month.f3                          0.000000e+00
month.f4                          0.000000e+00
month.f5                          0.000000e+00
month.f6                          0.000000e+00
month.f7                          0.000000e+00
month.f8                          0.000000e+00
month.f9                          0.000000e+00
month.f10                         0.000000e+00
month.f11                        8.973263e-251
month.f12                         2.063279e-08
log(TreeDiv, base = 2):month.f2   3.871123e-01
log(TreeDiv, base = 2):month.f3   1.958210e-01
log(TreeDiv, base = 2):month.f4   1.464016e-02
log(TreeDiv, base = 2):month.f5   5.027915e-03
log(TreeDiv, base = 2):month.f6   7.138252e-03
log(TreeDiv, base = 2):month.f7   6.868255e-04
log(TreeDiv, base = 2):month.f8   1.342109e-03
log(TreeDiv, base = 2):month.f9   1.006450e-02
log(TreeDiv, base = 2):month.f10  1.482667e-02
log(TreeDiv, base = 2):month.f11  6.919907e-02
log(TreeDiv, base = 2):month.f12  5.093336e-01

                               numDF denDF  F-value p-value
(Intercept)                        1  4079 3170.869  <.0001
log(TreeDiv, base = 2)             1    60    9.010  0.0039
month.f                           11  4079 1825.596  <.0001
log(TreeDiv, base = 2):month.f    11  4079    1.681  0.0713
```

Median temperature

```
mod.monthly.Tmed =
  lme(T.med ~ log(TreeDiv, base = 2) * month.f,
      random = ~ 1|site/plot/year,
      data = data,
      correlation=corCAR1(),
      na.action=na.exclude)
```

Model

Assumption validation

$NCV

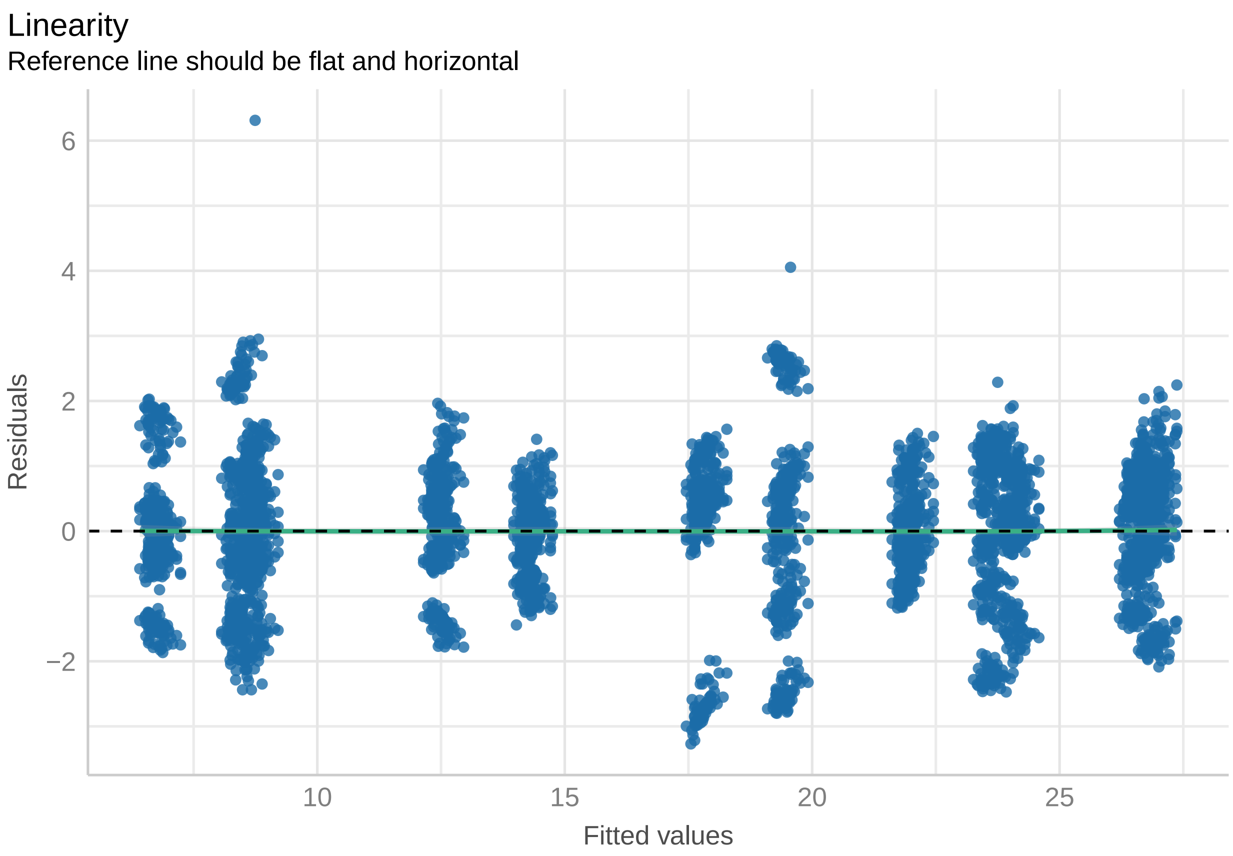

$VIF

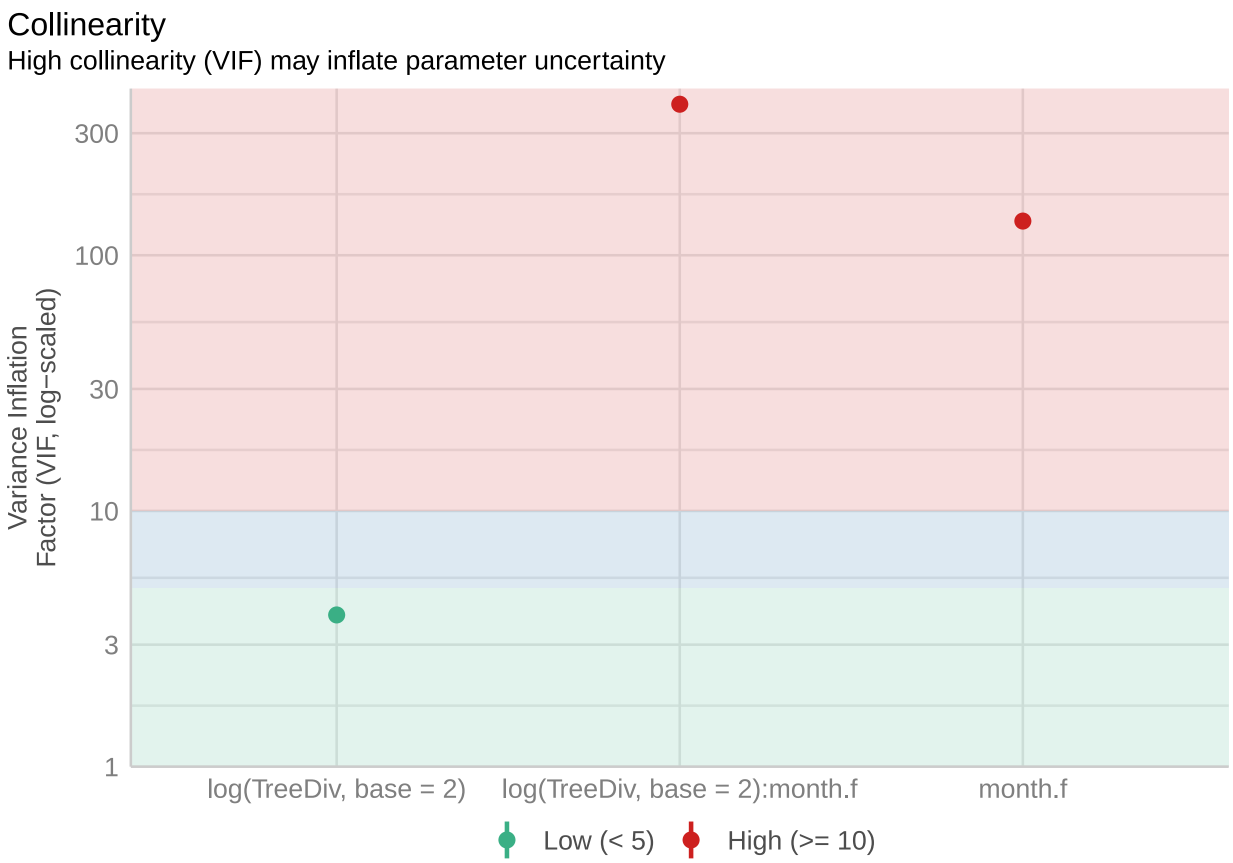
Collinearity
High collinearity (VIF) may inflate parameter uncertainty
Variance Inflation
Factor (VIF, log−scaled)
300
100
30
10
3
1
log(TreeDiv, base = 2)
log(TreeDiv, base = 2):month.f
month.f
Low (< 5)
High (>= 10)

```
$QQ
```

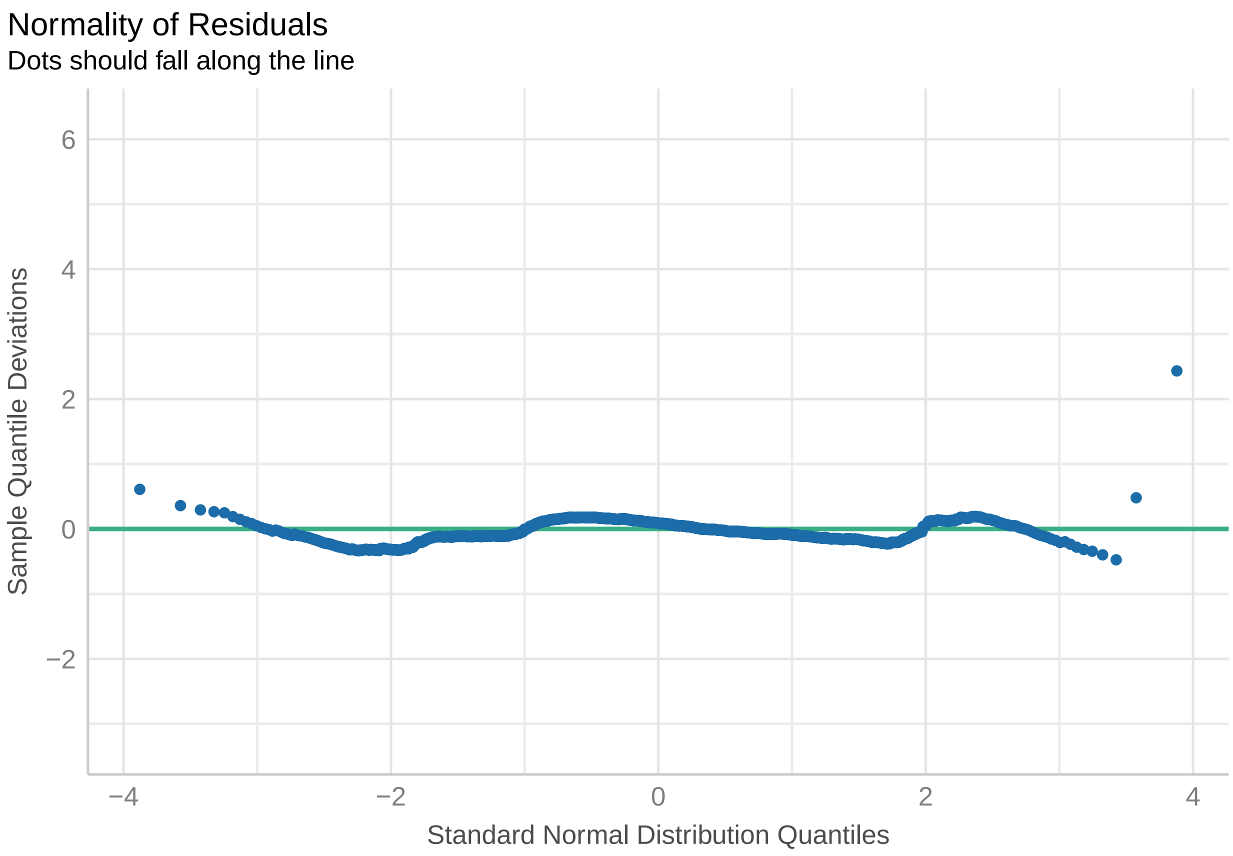
Normality of Residuals
Dots should fall along the line
Sample Quantile Deviations
6
4
2
0
−2
−4
−2
0
2
4
Standard Normal Distribution Quantiles

```
$NORM
```

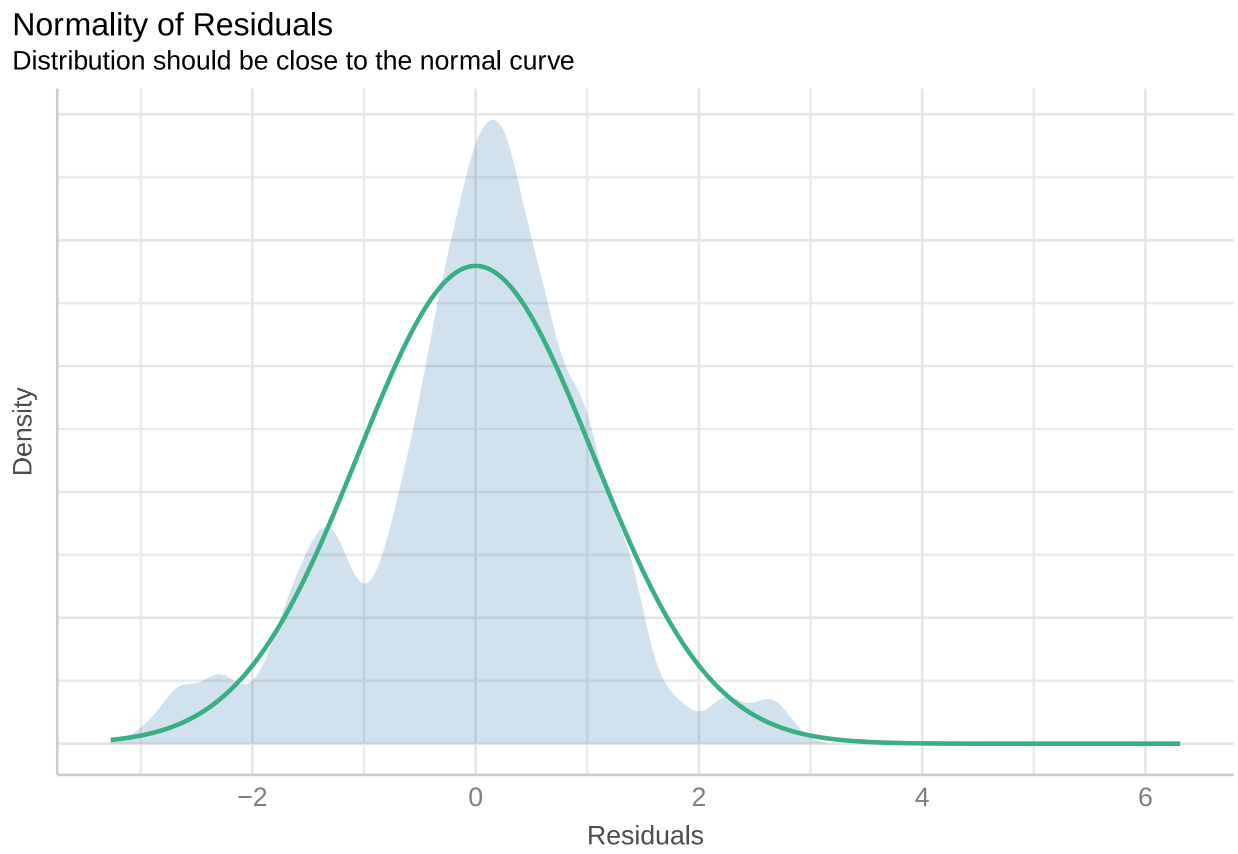

Summary

	Response	family	link	method	Marginal	Conditional
1	T.med	gaussian	identity	none	0.976606	0.9775269

	Value	Std.Error	DF	t-value
(Intercept)	6.768830462	0.10226932	4079	66.18632746
log(TreeDiv, base = 2)	-0.006701877	0.04773377	60	-0.14040118
month.f2	1.648061152	0.08921893	4079	18.47210234
month.f3	5.721374668	0.09601214	4079	59.59011942
month.f4	11.039676668	0.09684910	4079	113.98843328
month.f5	15.214060502	0.09701029	4079	156.82934744
month.f6	17.345918932	0.09703577	4079	178.75799540
month.f7	19.771210385	0.09703979	4079	203.74332397
month.f8	20.133788348	0.09704043	4079	207.47834547
month.f9	16.825512296	0.09707864	4079	173.31838007
month.f10	12.681436964	0.09717757	4079	130.49757351
month.f11	7.514031087	0.09717914	4079	77.32144284
month.f12	1.971003684	0.09714175	4079	20.28997574
log(TreeDiv, base = 2):month.f2	0.006319520	0.05669399	4079	0.11146719
log(TreeDiv, base = 2):month.f3	0.013335328	0.06101264	4079	0.21856663
log(TreeDiv, base = 2):month.f4	0.005368003	0.06162355	4079	0.08710961
log(TreeDiv, base = 2):month.f5	-0.011503335	0.06172640	4079	-0.18636006
log(TreeDiv, base = 2):month.f6	-0.008702268	0.06174265	4079	-0.14094418
log(TreeDiv, base = 2):month.f7	0.027324205	0.06174522	4079	0.44253149
log(TreeDiv, base = 2):month.f8	0.047749689	0.06174563	4079	0.77332905
log(TreeDiv, base = 2):month.f9	0.022842755	0.06174578	4079	0.36994842

```
log(TreeDiv, base = 2):month.f10  0.004725324 0.06176818 4079   0.07650094
log(TreeDiv, base = 2):month.f11  0.044895599 0.06176875 4079   0.72683359
log(TreeDiv, base = 2):month.f12  0.011412949 0.06176868 4079   0.18476919
                                       p-value
(Intercept)                       0.000000e+00
log(TreeDiv, base = 2)            8.888133e-01
month.f2                          3.114187e-73
month.f3                          0.000000e+00
month.f4                          0.000000e+00
month.f5                          0.000000e+00
month.f6                          0.000000e+00
month.f7                          0.000000e+00
month.f8                          0.000000e+00
month.f9                          0.000000e+00
month.f10                         0.000000e+00
month.f11                         0.000000e+00
month.f12                         2.802751e-87
log(TreeDiv, base = 2):month.f2   9.112514e-01
log(TreeDiv, base = 2):month.f3   8.269986e-01
log(TreeDiv, base = 2):month.f4   9.305887e-01
log(TreeDiv, base = 2):month.f5   8.521717e-01
log(TreeDiv, base = 2):month.f6   8.879210e-01
log(TreeDiv, base = 2):month.f7   6.581281e-01
log(TreeDiv, base = 2):month.f8   4.393725e-01
log(TreeDiv, base = 2):month.f9   7.114401e-01
log(TreeDiv, base = 2):month.f10  9.390243e-01
log(TreeDiv, base = 2):month.f11  4.673696e-01
log(TreeDiv, base = 2):month.f12  8.534193e-01

                                numDF denDF  F-value p-value
(Intercept)                         1  4079 52216.51  <.0001
log(TreeDiv, base = 2)              1    60     0.01  0.9327
month.f                            11  4079 14424.28  <.0001
log(TreeDiv, base = 2):month.f     11  4079     0.18  0.9984
```

Minimum temperature

```
mod.monthly.Tmin =
  lme(T.min ~ log(TreeDiv, base = 2) * month.f,
      random = ~ 1|site/plot/year,
      data = data,
      correlation=corCAR1(),
      na.action=na.exclude)
```

Model

Assumption validation

$NCV

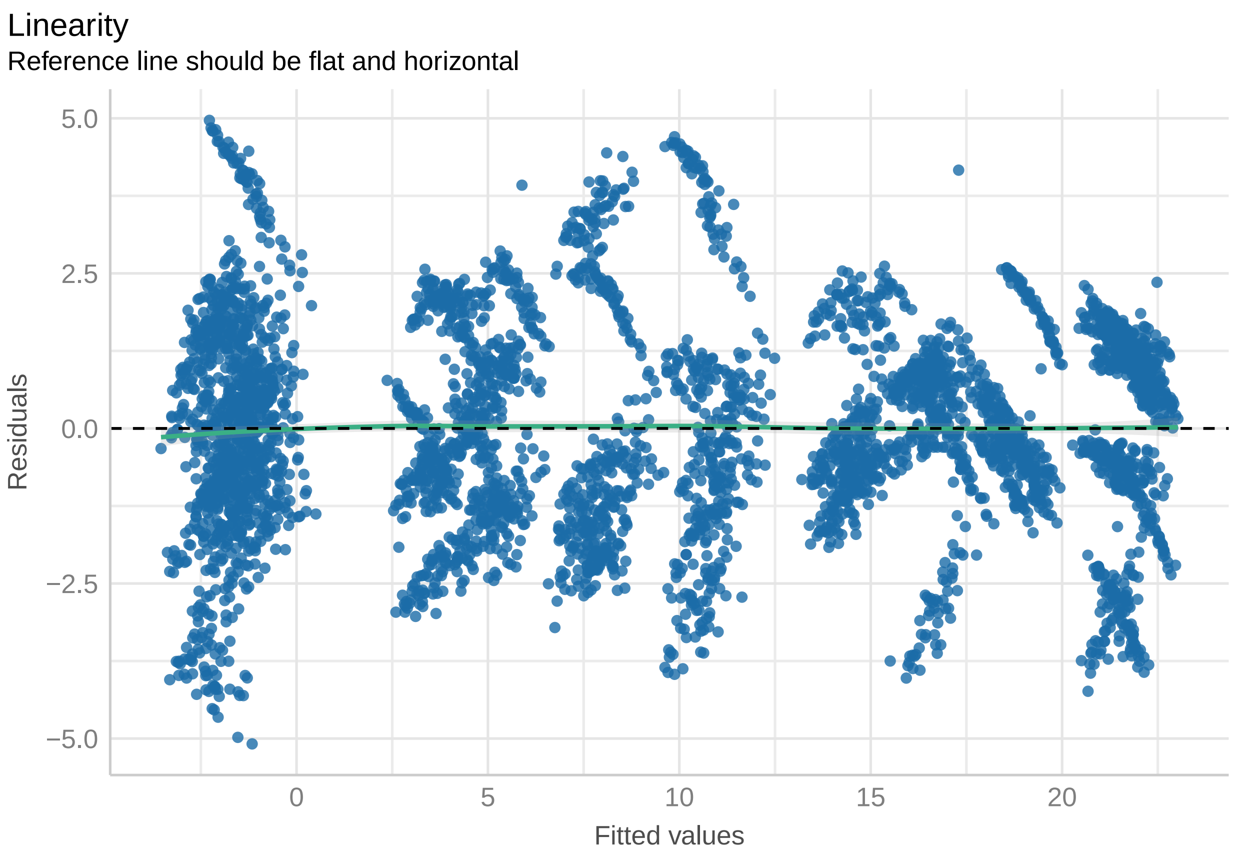

$VIF

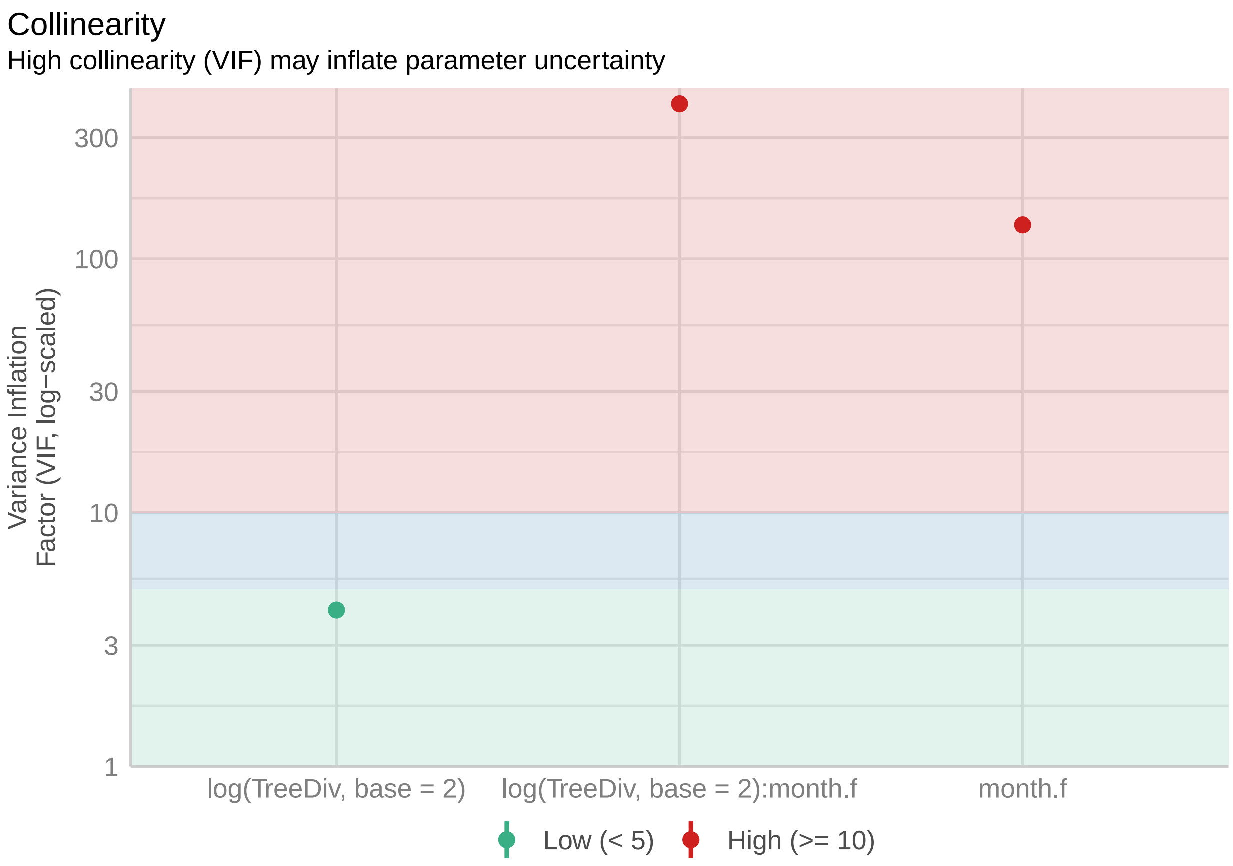
Collinearity
High collinearity (VIF) may inflate parameter uncertainty
Variance Inflation
Factor (VIF, log−scaled)
300
100
30
10
3
1
log(TreeDiv, base = 2)
log(TreeDiv, base = 2):month.f
month.f
Low (< 5)
High (>= 10)

$QQ

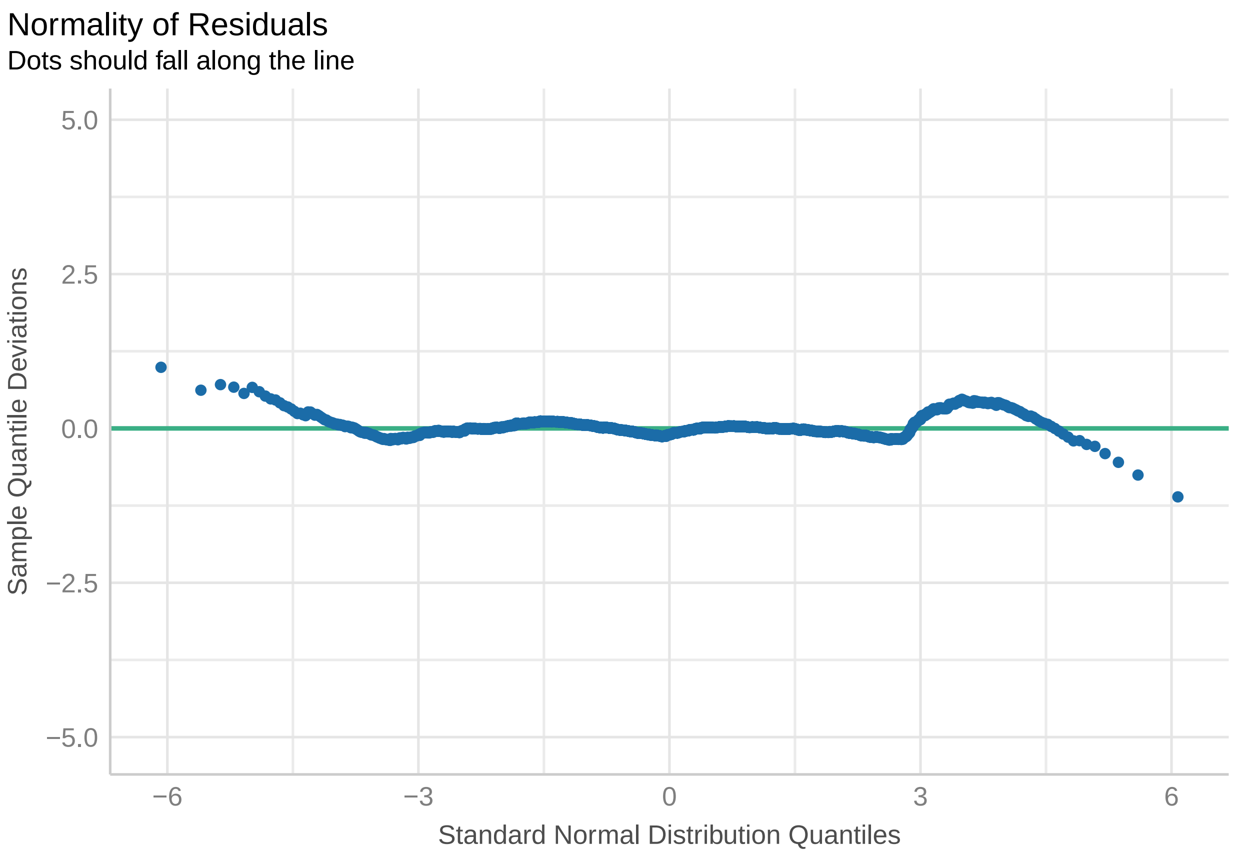
Normality of Residuals
Dots should fall along the line
Sample Quantile Deviations
5.0
2.5
0.0
−2.5
−5.0
−6
−3
0
3
6
Standard Normal Distribution Quantiles

```
$NORM
```

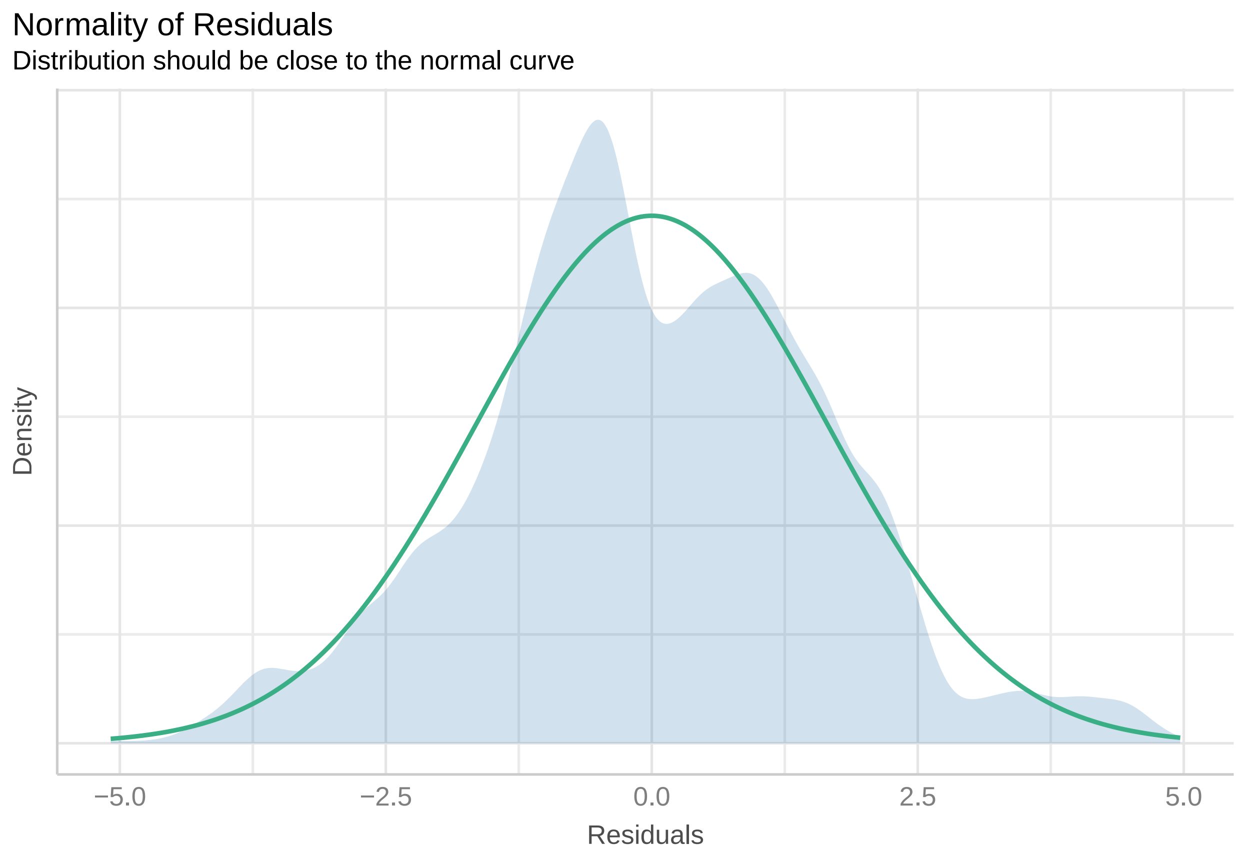

Summary

```
  Response   family     link method Marginal Conditional
1    T.min gaussian identity   none 0.956104   0.9631435

                                      Value  Std.Error   DF     t-value
(Intercept)                    -2.382067736 0.39538293 4079  -6.0247107
log(TreeDiv, base = 2)          0.230373627 0.07574149   60   3.0415776
month.f2                        0.651024277 0.14434421 4079   4.5102209
month.f3                        5.908516812 0.15379733 4079  38.4175518
month.f4                       10.122382156 0.15473265 4079  65.4185278
month.f5                       16.742223652 0.15489810 4079 108.0854018
month.f6                       21.178165737 0.15492048 4079 136.7034581
month.f7                       24.213576294 0.15492351 4079 156.2937480
month.f8                       23.817736338 0.15492392 4079 153.7382742
month.f9                       18.857430155 0.15498715 4079 121.6709273
month.f10                      12.938571364 0.15515341 4079  83.3921189
month.f11                       7.451452399 0.15515712 4079  48.0252030
month.f12                       0.944843400 0.15510123 4079   6.0917852
log(TreeDiv, base = 2):month.f2  0.007844573 0.09172322 4079   0.0855244
log(TreeDiv, base = 2):month.f3 -0.080967219 0.09773236 4079  -0.8284586
log(TreeDiv, base = 2):month.f4 -0.034794391 0.09844868 4079  -0.3534267
log(TreeDiv, base = 2):month.f5 -0.033229754 0.09855425 4079  -0.3371722
log(TreeDiv, base = 2):month.f6 -0.193409268 0.09856853 4079  -1.9621807
log(TreeDiv, base = 2):month.f7 -0.200310608 0.09857046 4079  -2.0321565
log(TreeDiv, base = 2):month.f8 -0.109700041 0.09857072 4079  -1.1129069
log(TreeDiv, base = 2):month.f9 -0.026438142 0.09857090 4079  -0.2682145
```

```
log(TreeDiv, base = 2):month.f10  0.015154855 0.09860853 4079   0.1536871
log(TreeDiv, base = 2):month.f11 -0.072531542 0.09860959 4079  -0.7355425
log(TreeDiv, base = 2):month.f12  0.017238217 0.09860954 4079   0.1748129
                                      p-value
(Intercept)                      1.843779e-09
log(TreeDiv, base = 2)           3.487242e-03
month.f2                         6.658050e-06
month.f3                        6.916878e-276
month.f4                         0.000000e+00
month.f5                         0.000000e+00
month.f6                         0.000000e+00
month.f7                         0.000000e+00
month.f8                         0.000000e+00
month.f9                         0.000000e+00
month.f10                        0.000000e+00
month.f11                        0.000000e+00
month.f12                        1.219753e-09
log(TreeDiv, base = 2):month.f2   9.318487e-01
log(TreeDiv, base = 2):month.f3   4.074593e-01
log(TreeDiv, base = 2):month.f4   7.237868e-01
log(TreeDiv, base = 2):month.f5   7.360044e-01
log(TreeDiv, base = 2):month.f6   4.980934e-02
log(TreeDiv, base = 2):month.f7   4.220248e-02
log(TreeDiv, base = 2):month.f8   2.658140e-01
log(TreeDiv, base = 2):month.f9   7.885478e-01
log(TreeDiv, base = 2):month.f10  8.778641e-01
log(TreeDiv, base = 2):month.f11  4.620515e-01
log(TreeDiv, base = 2):month.f12  8.612353e-01

                               numDF denDF  F-value p-value
(Intercept)                        1  4079  613.346  <.0001
log(TreeDiv, base = 2)             1    60   18.807  0.0001
month.f                           11  4079 8791.357  <.0001
log(TreeDiv, base = 2):month.f    11  4079    1.093  0.3620
```

Fig. 2

Output S4: Fig. 2.A. Monthly buffering model structure and outputs

```
mod.monthly.buff = lme(T.buff ~ log(TreeDiv, base = 2) * month.f,
              random = ~ 1|site/plot/year,
              data = d.2.2,
              correlation=corCAR1(form = ~year.month),
              na.action=na.exclude)
```

Model

Assumption validation

$NCV

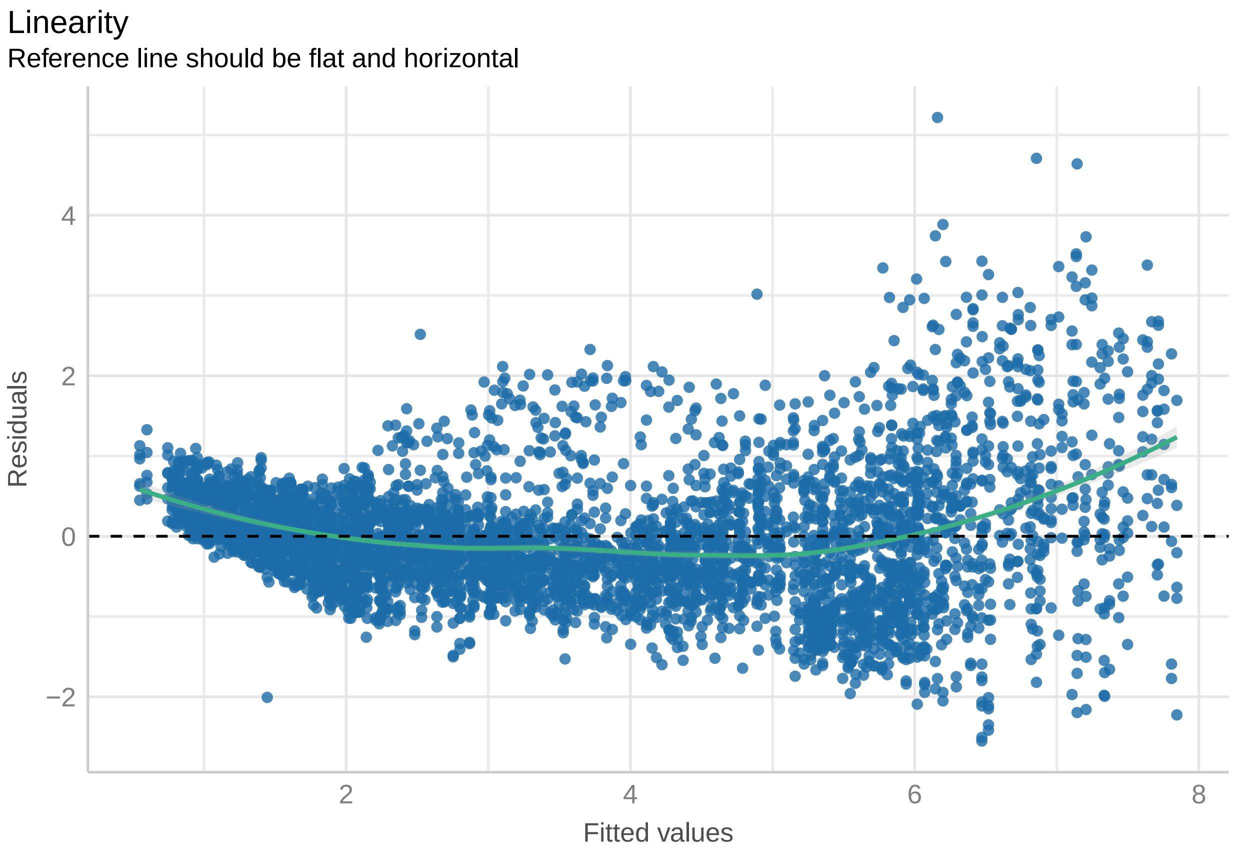

$VIF

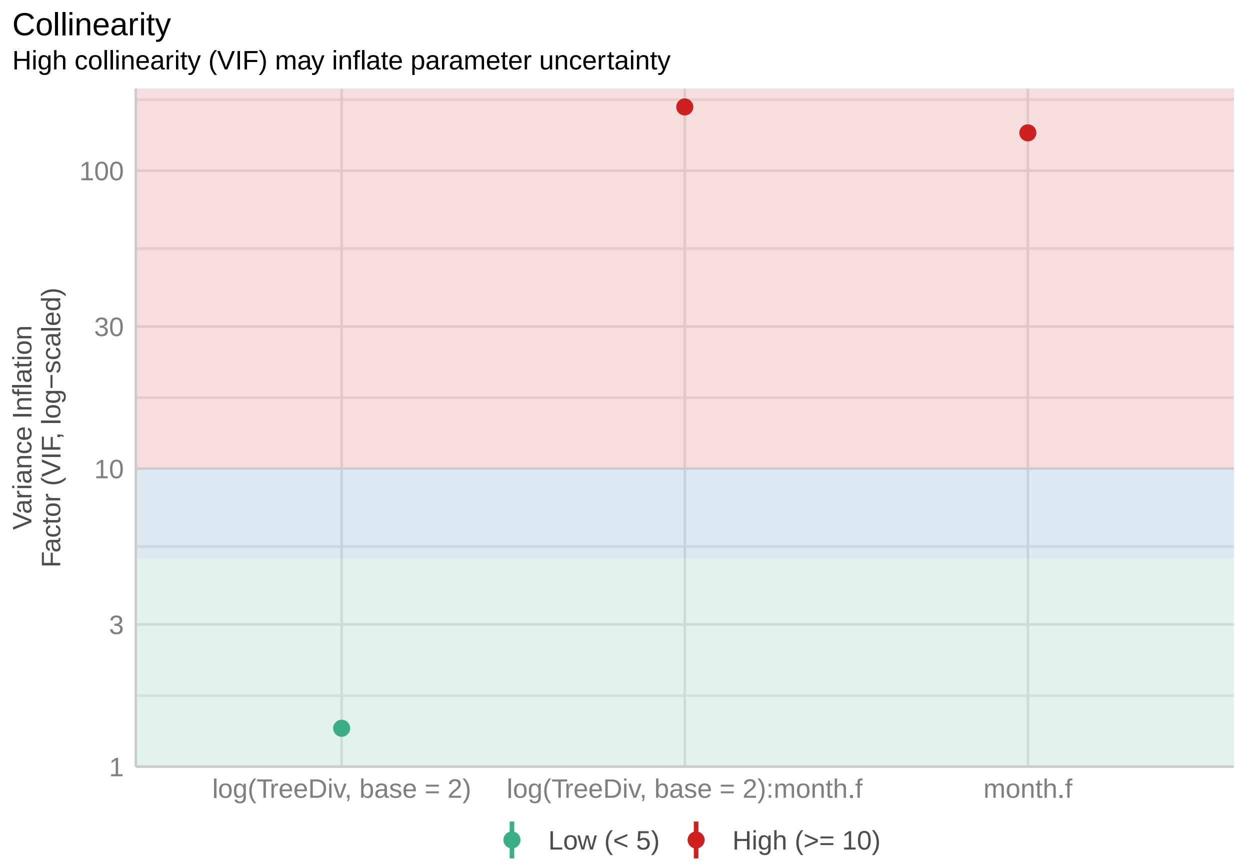
Collinearity
High collinearity (VIF) may inflate parameter uncertainty
Variance Inflation Factor (VIF, log−scaled)
100
30
10
3
1
log(TreeDiv, base = 2)
log(TreeDiv, base = 2):month.f
month.f
Low (< 5)
High (>= 10)

```
$QQ
```

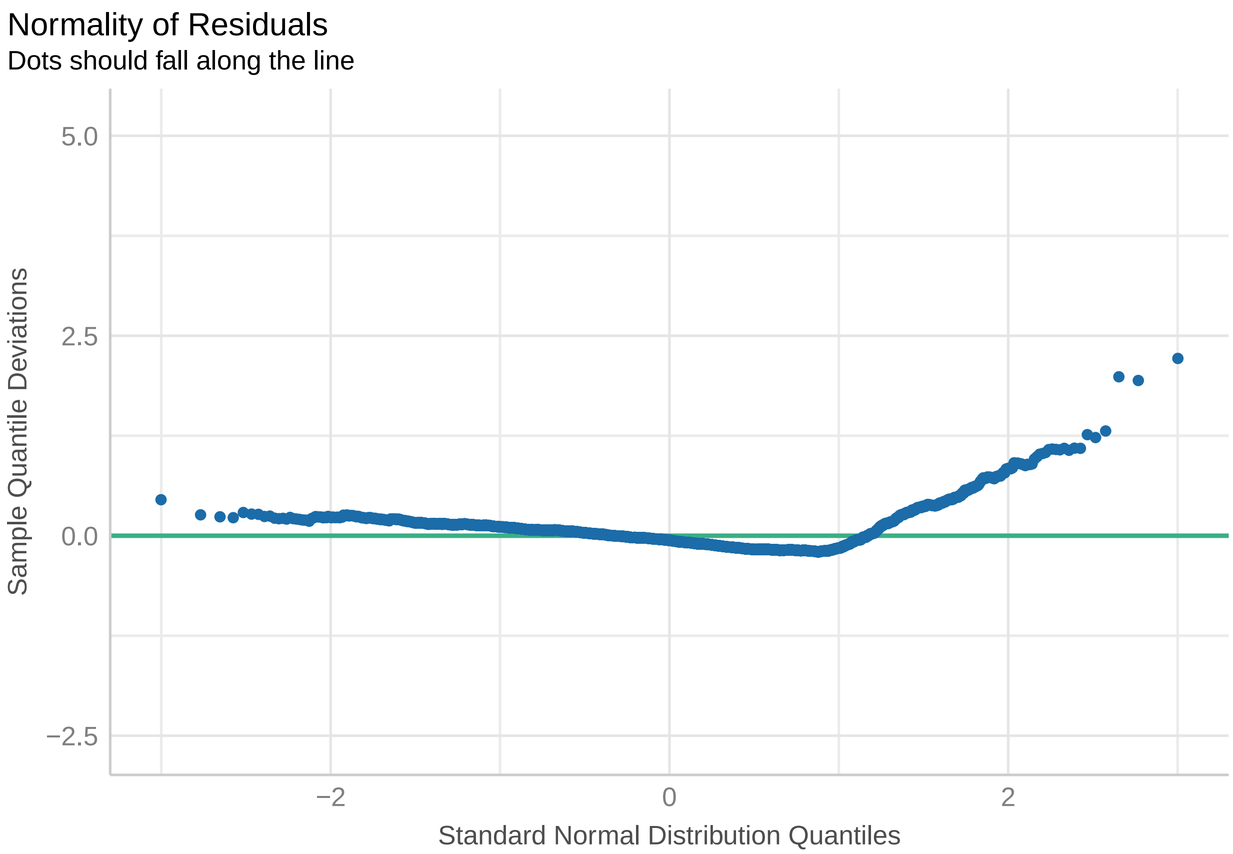
Normality of Residuals
Dots should fall along the line
Sample Quantile Deviations
5.0
2.5
0.0
−2.5
−2
0
2
Standard Normal Distribution Quantiles

$NORM

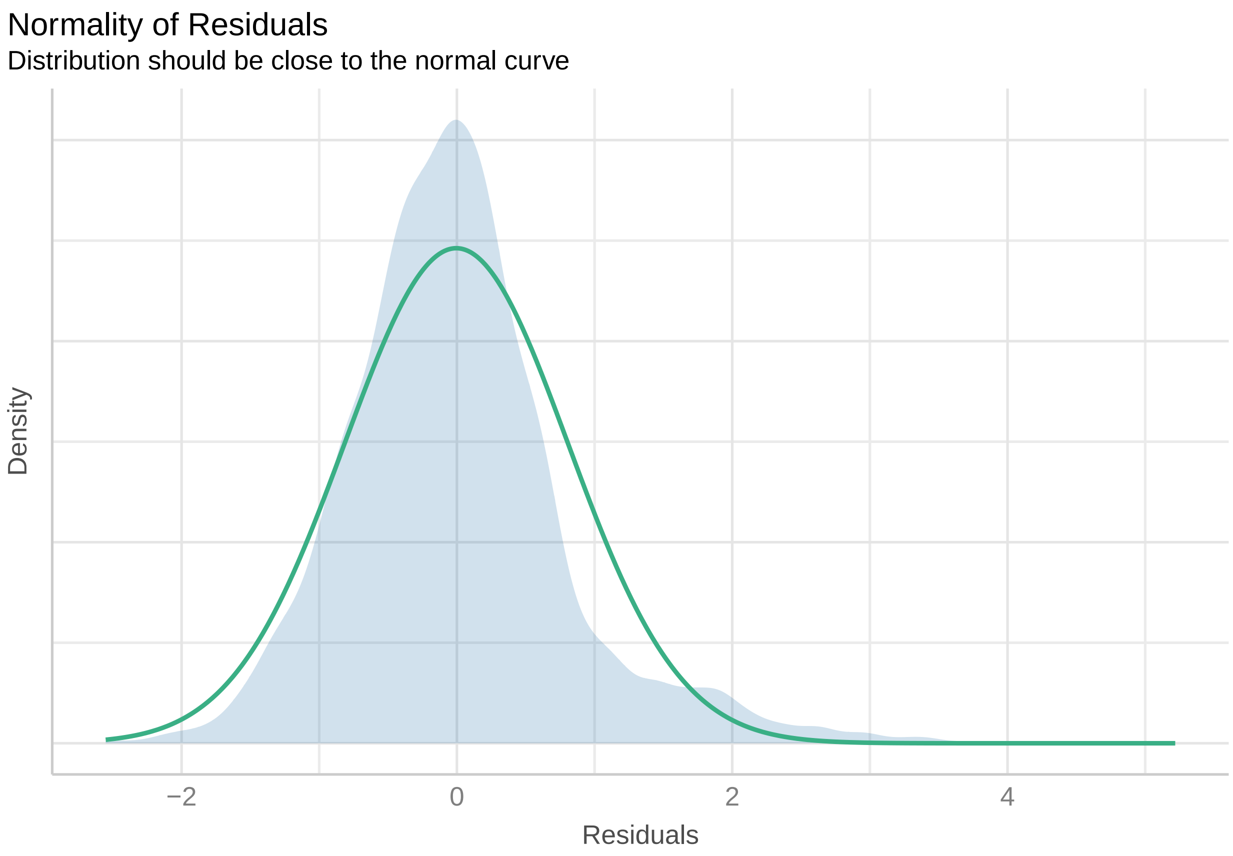

Summary

```
  Response   family     link method  Marginal Conditional
1   T.buff gaussian identity   none 0.7746175   0.8466192

                                       Value  Std.Error   DF     t-value
(Intercept)                       4.80205227 0.26169456 4279  18.3498357
log(TreeDiv, base = 2)            0.25350399 0.05316338   60   4.7683948
month.f10                        -1.18125521 0.04810778 4279 -24.5543503
month.f11                        -2.12810299 0.05902780 4279 -36.0525529
month.f12                        -3.26147523 0.06383239 4279 -51.0943645
month.f1                         -3.37666446 0.07117618 4279 -47.4409319
month.f2                         -3.32994547 0.07102882 4279 -46.8816102
month.f3                         -2.60253399 0.07072187 4279 -36.7995664
month.f4                         -1.85799722 0.07005255 4279 -26.5229072
month.f5                         -0.24416816 0.06895336 4279  -3.5410624
month.f6                          1.06990751 0.06671138 4279  16.0378549
month.f7                          1.11648075 0.06197032 4279  18.0163794
month.f8                          1.00701378 0.05097758 4279  19.7540523
log(TreeDiv, base = 2):month.f10 -0.09530291 0.03065742 4279  -3.1086411
log(TreeDiv, base = 2):month.f11 -0.16892082 0.03762432 4279  -4.4896706
log(TreeDiv, base = 2):month.f12 -0.19576952 0.04069915 4279  -4.8101625
log(TreeDiv, base = 2):month.f1  -0.21613310 0.04534123 4279  -4.7668114
log(TreeDiv, base = 2):month.f2  -0.21522325 0.04525062 4279  -4.7562496
log(TreeDiv, base = 2):month.f3  -0.19619711 0.04506759 4279  -4.3533972
log(TreeDiv, base = 2):month.f4  -0.14581015 0.04468991 4279  -3.2627084
log(TreeDiv, base = 2):month.f5  -0.01009822 0.04398798 4279  -0.2295677
```

```
log(TreeDiv, base = 2):month.f6   0.05275429 0.04255628 4279   1.2396359
log(TreeDiv, base = 2):month.f7   0.15338392 0.03952874 4279   3.8803137
log(TreeDiv, base = 2):month.f8   0.16907377 0.03250894 4279   5.2008386
                                       p-value
(Intercept)                       1.864953e-72
log(TreeDiv, base = 2)            1.224900e-05
month.f10                        1.141670e-124
month.f11                        8.655202e-249
month.f12                         0.000000e+00
month.f1                          0.000000e+00
month.f2                          0.000000e+00
month.f3                         8.133152e-258
month.f4                         1.217975e-143
month.f5                          4.027646e-04
month.f6                          2.944591e-56
month.f7                          5.276874e-70
month.f8                          3.405283e-83
log(TreeDiv, base = 2):month.f10  1.891841e-03
log(TreeDiv, base = 2):month.f11  7.321040e-06
log(TreeDiv, base = 2):month.f12  1.559950e-06
log(TreeDiv, base = 2):month.f1   1.933789e-06
log(TreeDiv, base = 2):month.f2   2.037147e-06
log(TreeDiv, base = 2):month.f3   1.371754e-05
log(TreeDiv, base = 2):month.f4   1.112193e-03
log(TreeDiv, base = 2):month.f5   8.184387e-01
log(TreeDiv, base = 2):month.f6   2.151781e-01
log(TreeDiv, base = 2):month.f7   1.058914e-04
log(TreeDiv, base = 2):month.f8   2.076623e-07

                                numDF denDF   F-value p-value
(Intercept)                         1  4279  179.5570  <.0001
log(TreeDiv, base = 2)              1    60   11.9622   0.001
month.f                            11  4279 1287.5399  <.0001
log(TreeDiv, base = 2):month.f     11  4279   11.5438  <.0001
```

Output S5: Fig. 2.C. Yearly model structure and outputs

```
mod.yearly.buff =
  lme(T.buff ~ log(TreeDiv, base = 2) * year,
      random = ~ 1|site/plot,
      data = d.1.2,
      correlation=corCAR1(form = ~year),
      na.action=na.exclude)
```

Model

Assumption validation

```
$NCV
```

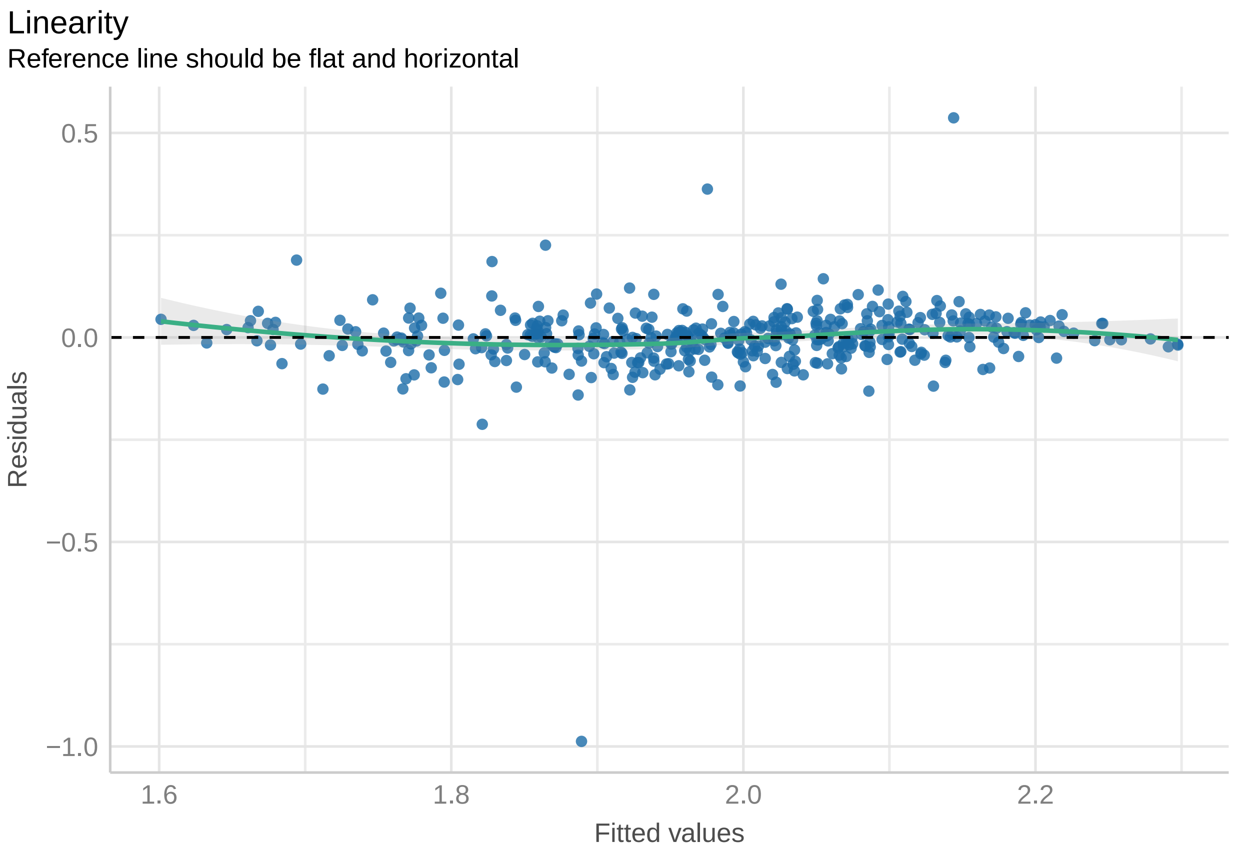


```
$VIF
```

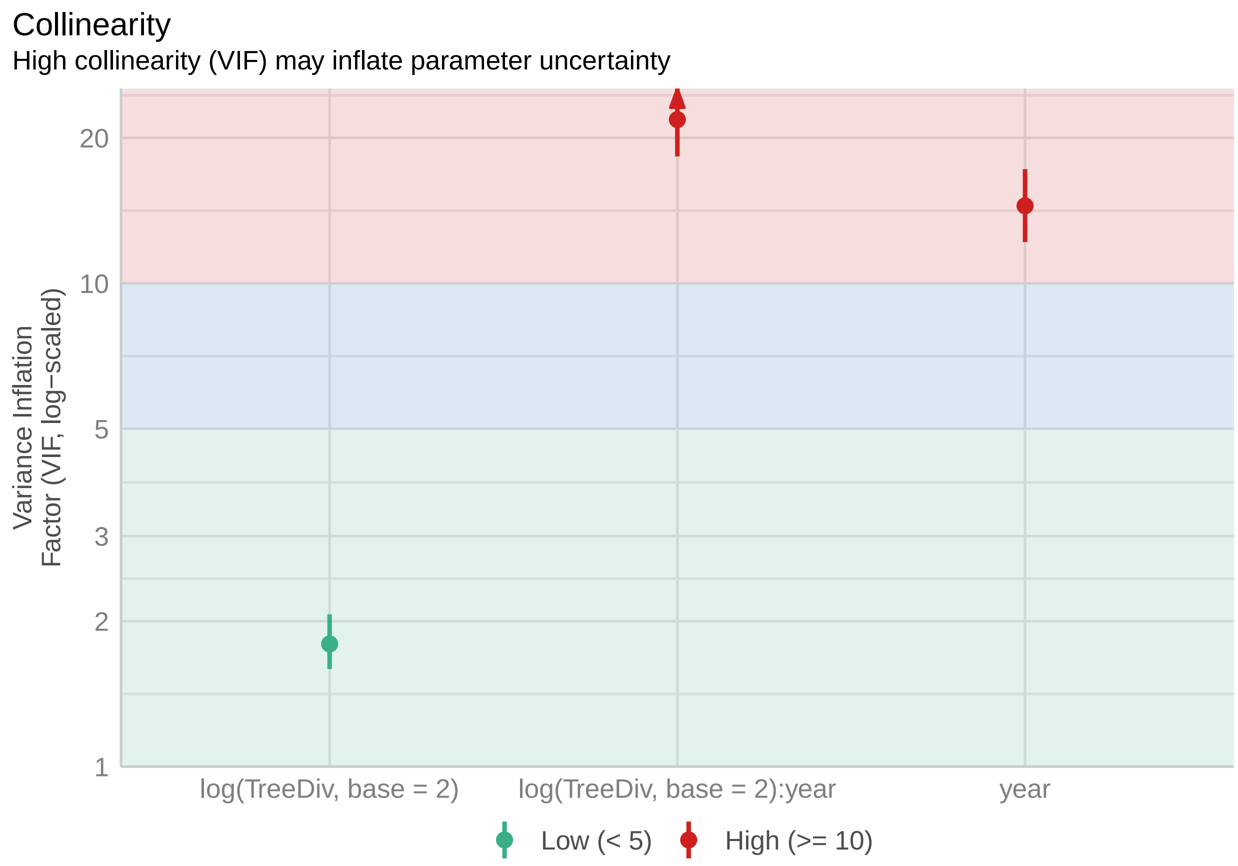
Collinearity
High collinearity (VIF) may inflate parameter uncertainty
Variance Inflation
Factor (VIF, log−scaled)
20
10
5
3
2
1
log(TreeDiv, base = 2)
log(TreeDiv, base = 2):year
year
Low (< 5)
High (>= 10)

$QQ

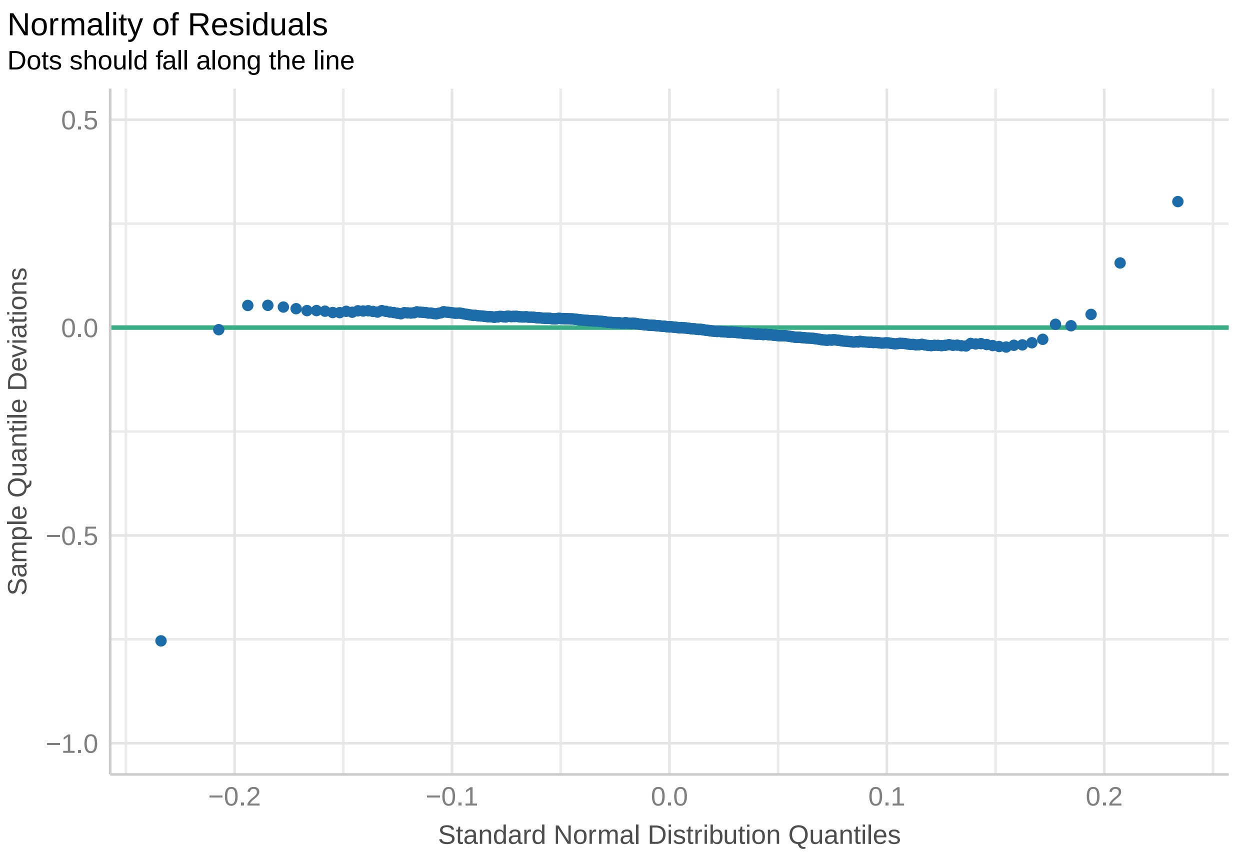
Normality of Residuals
Dots should fall along the line
0.5
0.0
−0.5
−1.0
Sample Quantile Deviations
−0.2
−0.1
0.0
0.1
0.2
Standard Normal Distribution Quantiles

```
$NORM
```

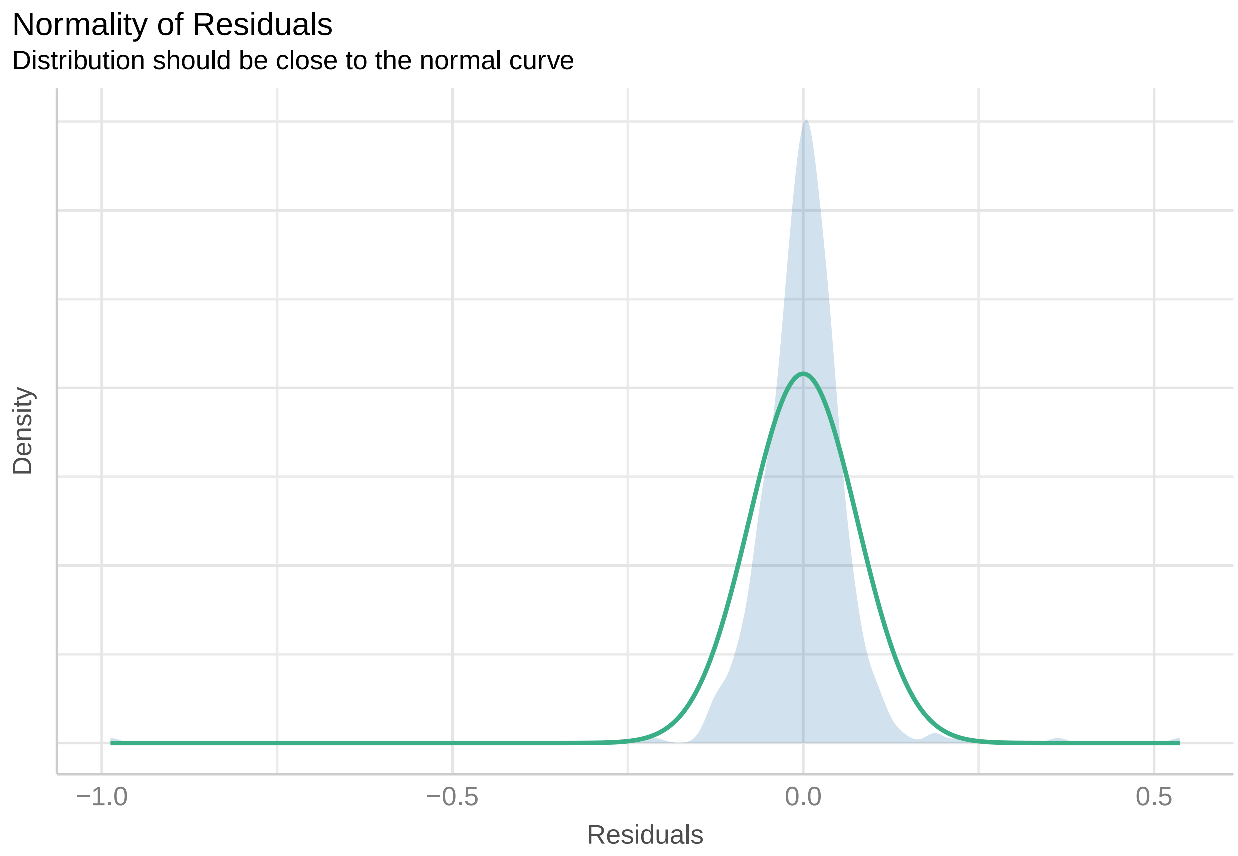

Summary

```
  Response   family     link method  Marginal Conditional
1   T.buff gaussian identity   none 0.3854403   0.7582944

                                        Value  Std.Error  DF     t-value
(Intercept)                       1.762007797 0.05936524 364 29.68079835
log(TreeDiv, base = 2)            0.029614524 0.01151735  60  2.57129648
year2015                          0.294044446 0.01607911 364 18.28735732
year2016                          0.191541748 0.01847229 364 10.36913937
year2017                          0.226636013 0.01917476 364 11.81949790
year2018                          0.092814552 0.01943908 364  4.77463809
year2019                          0.263262284 0.01950853 364 13.49472551
year2020                          0.287903142 0.01948589 364 14.77495663
log(TreeDiv, base = 2):year2015 -0.003286320 0.01028277 364 -0.31959470
log(TreeDiv, base = 2):year2016 -0.004185857 0.01181324 364 -0.35433614
log(TreeDiv, base = 2):year2017  0.004006050 0.01226247 364  0.32669178
log(TreeDiv, base = 2):year2018 -0.001227485 0.01240283 364 -0.09896813
log(TreeDiv, base = 2):year2019 -0.007948457 0.01244735 364 -0.63856596
log(TreeDiv, base = 2):year2020  0.016124927 0.01246145 364  1.29398529
                                     p-value
(Intercept)                     3.151520e-99
log(TreeDiv, base = 2)          1.262846e-02
year2015                        1.862496e-53
year2016                        3.032205e-22
year2017                        1.665762e-27
year2018                        2.613085e-06
```

```
year2019                         6.209248e-34
year2020                         4.965998e-39
log(TreeDiv, base = 2):year2015 7.494589e-01
log(TreeDiv, base = 2):year2016 7.232922e-01
log(TreeDiv, base = 2):year2017 7.440888e-01
log(TreeDiv, base = 2):year2018 9.212181e-01
log(TreeDiv, base = 2):year2019 5.235069e-01
log(TreeDiv, base = 2):year2020 1.964909e-01

                            numDF denDF   F-value p-value
(Intercept)                     1   364 1181.4544  <.0001
log(TreeDiv, base = 2)          1    60   13.0130  0.0006
year                            6   364  139.7500  <.0001
log(TreeDiv, base = 2):year     6   364    1.0842  0.3713
```

Output S6: SPEI model structure and outputs

```
mod.spei = lme(T.buff ~ log(TreeDiv, base = 2) * spei,
              random = ~ 1|site/plot,
              data = d.1.3,
              correlation=corCAR1(form = ~year),
              na.action=na.exclude)
```

Model structure

Model output

```
                                   Value   Std.Error  DF     t-value
(Intercept)                 1.953790e+00 0.059747279 311 32.70090930
log(TreeDiv, base = 2)      3.017577e-02 0.009120243  60  3.30865858
spei                        5.329209e-02 0.007266291 311  7.33415286
log(TreeDiv, base = 2):spei 5.303695e-05 0.004637089 311  0.01143755
                                  p-value
(Intercept)                 1.164624e-102
log(TreeDiv, base = 2)       1.588329e-03
spei                         1.948952e-12
log(TreeDiv, base = 2):spei  9.908817e-01
```

Figure S12: comparison CRU and local measurements

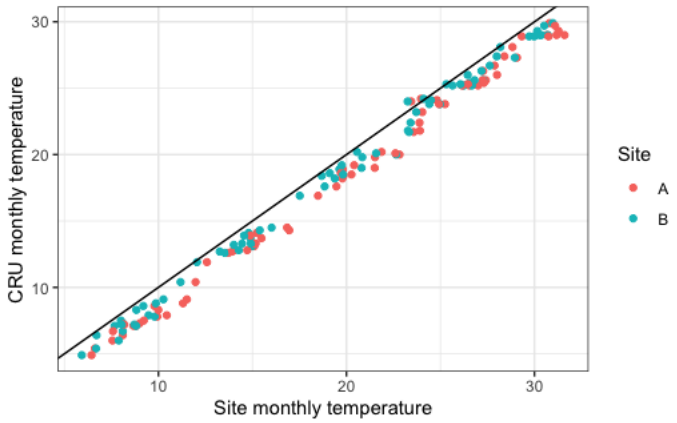

Comparison between monthly temperature averages from the CRU dataset and local temperature measurements from HOBO loggers on bare plots without trees (n = 6) during the study period 2015–2020. The black line shows a 1:1 line and coloured points show the data comparison separated according to experimental site (A and B). Note that our random effects structure controls for differences between the two sites.

SEM models outputs

Output S7: yearly scale SEM output

```
Structural Equation Model of mod.sem

Call:
  Buff.cor ~ mean.height + l.SSCI + LAI + l.TreeDiv
  l.SSCI ~ l.TreeDiv
  LAI ~ l.TreeDiv + KoBi + ScSu + QuFa
  mean.height ~ l.TreeDiv
  LAI ~~ l.SSCI
  mean.height ~~ l.SSCI
  mean.height ~~ LAI

    AIC
 1692.479

---
Tests of directed separation:

            Independ.Claim Test.Type DF Crit.Value P.Value
     Buff.cor ~ KoBi + ...      coef 26     0.0072  0.9943
  mean.height ~ KoBi + ...      coef 29    -1.3678  0.1819
       l.SSCI ~ KoBi + ...      coef 29     1.5580  0.1301
     Buff.cor ~ ScSu + ...      coef 26    -0.8666  0.3941
  mean.height ~ ScSu + ...      coef 29     1.2918  0.2066
       l.SSCI ~ ScSu + ...      coef 29    -0.1000  0.9210
     Buff.cor ~ QuFa + ...      coef 26     0.9008  0.3760
  mean.height ~ QuFa + ...      coef 29    -0.9980  0.3265
       l.SSCI ~ QuFa + ...      coef 29     1.0003  0.3254

--
Global goodness-of-fit:

Chi-Squared = 892.389 with P-value = 0 and on 9 degrees of freedom
Fisher's C = 19.121 with P-value = 0.384 and on 18 degrees of freedom

---
Coefficients:

       Response   Predictor Estimate Std.Error DF Crit.Value P.Value
       Buff.cor mean.height   0.0705    0.1061 22     0.6643  0.5134
       Buff.cor      l.SSCI   0.2224    0.0634 22     3.5105  0.0020
       Buff.cor         LAI   0.3097    0.1134 22     2.7306  0.0122
       Buff.cor   l.TreeDiv   0.0705     0.071 22     0.9936  0.3312
         l.SSCI   l.TreeDiv   0.1712    0.0551 72     3.1062  0.0027
            LAI   l.TreeDiv   0.8205    0.2915 49     2.8145  0.0070
            LAI        KoBi  -2.7238    0.8189 49    -3.3261  0.0017
            LAI        ScSu   3.1619    0.8475 49     3.7309  0.0005
            LAI        QuFa  -2.0357    0.9036 49    -2.2530  0.0288
    mean.height   l.TreeDiv  -3.0058   30.2082 30    -0.0995  0.9214
          ~~LAI    ~~l.SSCI   0.1140         - 74     0.9672  0.1684
```

```
  ~~mean.height    ~~l.SSCI  -0.1892         - 74    -1.6233  0.0545
  ~~mean.height       ~~LAI   0.2235         - 54     1.6379  0.0538
  Std.Estimate
       14.7843
        0.1491  **
        0.7262   *
        0.0894
        0.3236  **
        0.4434  **
       -0.4285  **
        0.4974 ***
       -0.3202   *
       -0.0182
        0.1140
       -0.1892
        0.2235

  Signif. codes:  0 '***' 0.001 '**' 0.01 '*' 0.05

---
Individual R-squared:

     Response method Marginal Conditional
     Buff.cor   none     0.17        0.91
       l.SSCI   none     0.12        0.89
          LAI   none     0.37        0.92
  mean.height   none     0.00        0.88
```

Output S8: Monthly SEM model outputs

Month	Response	Predictor	Estimate	Std.Error	DF	P.Value	
1	Buff.cor	Mean height	-0.1557	0.2401	21	0.5235	
1	Buff.cor	SSCI	0.2712	0.1426	21	0.0710	
1	Buff.cor	LAI	0.5189	0.2495	21	0.0500	*
1	Buff.cor	Tree sp. richness	-0.0568	0.1499	21	0.7087	
2	Buff.cor	Mean height	0.0292	0.1192	21	0.8086	
2	Buff.cor	SSCI	0.1513	0.0708	21	0.0445	*
2	Buff.cor	LAI	0.1572	0.1239	21	0.2184	
2	Buff.cor	Tree sp. richness	-0.0626	0.0744	21	0.4096	
3	Buff.cor	Mean height	-0.0936	0.0901	21	0.3110	
3	Buff.cor	SSCI	0.0971	0.0535	21	0.0840	
3	Buff.cor	LAI	0.2337	0.0936	21	0.0209	*
3	Buff.cor	Tree sp. richness	-0.0212	0.0562	21	0.7105	
4	Buff.cor	Mean height	0.0215	0.0636	22	0.7386	
4	Buff.cor	SSCI	0.0971	0.038	22	0.0180	*
4	Buff.cor	LAI	0.1978	0.0682	22	0.0083	**
4	Buff.cor	Tree sp. richness	0.0092	0.043	22	0.8332	
5	Buff.cor	Mean height	0.1409	0.1355	22	0.3099	
5	Buff.cor	SSCI	0.1650	0.081	22	0.0538	
5	Buff.cor	LAI	0.3809	0.1455	22	0.0157	*
5	Buff.cor	Tree sp. richness	0.1416	0.0917	22	0.1368	
6	Buff.cor	Mean height	0.1712	0.1874	22	0.3710	
6	Buff.cor	SSCI	0.2159	0.112	22	0.0668	
6	Buff.cor	LAI	0.5181	0.2012	22	0.0173	*
6	Buff.cor	Tree sp. richness	0.1158	0.1268	22	0.3707	
7	Buff.cor	Mean height	0.1505	0.1729	22	0.3935	
7	Buff.cor	SSCI	0.2801	0.1033	22	0.0128	*
7	Buff.cor	LAI	0.5051	0.1856	22	0.0125	*
7	Buff.cor	Tree sp. richness	0.0904	0.1169	22	0.4476	
8	Buff.cor	Mean height	0.1427	0.1677	22	0.4039	
8	Buff.cor	SSCI	0.3154	0.1002	22	0.0047	**
8	Buff.cor	LAI	0.5858	0.1801	22	0.0036	**
8	Buff.cor	Tree sp. richness	0.1764	0.1134	22	0.1343	
9	Buff.cor	Mean height	0.0639	0.1334	22	0.6367	
9	Buff.cor	SSCI	0.2537	0.0797	22	0.0043	**
9	Buff.cor	LAI	0.3615	0.1433	22	0.0193	*
9	Buff.cor	Tree sp. richness	0.1464	0.0902	22	0.1189	
10	Buff.cor	Mean height	0.0511	0.0909	22	0.5799	
10	Buff.cor	SSCI	0.1791	0.0543	22	0.0033	**
10	Buff.cor	LAI	0.1770	0.0976	22	0.0834	
10	Buff.cor	Tree sp. richness	0.0799	0.0615	22	0.2071	
11	Buff.cor	Mean height	-0.0381	0.1153	22	0.7444	
11	Buff.cor	SSCI	0.1883	0.0689	22	0.0121	*
11	Buff.cor	LAI	0.2286	0.1238	22	0.0784	
11	Buff.cor	Tree sp. richness	0.0366	0.078	22	0.6433	
12	Buff.cor	Mean height	-0.0780	0.121	22	0.5260	
12	Buff.cor	SSCI	0.2006	0.0723	22	0.0111	*
12	Buff.cor	LAI	0.2352	0.1299	22	0.0838	
12	Buff.cor	Tree sp. richness	0.0563	0.0818	22	0.4985	

Output S9: SEM model using tree basal area, model structure and outputs.

```
Structural Equation Model of mod.sem

Call:
  Buff.cor ~ mean.height + l.SSCI + BA + l.TreeDiv
  l.SSCI ~ l.TreeDiv
  BA ~ l.TreeDiv + KoBi + ScSu + QuFa
  mean.height ~ l.TreeDiv
  BA ~~ l.SSCI
  mean.height ~~ l.SSCI
  mean.height ~~ BA

    AIC
 2850.993

---
Tests of directed separation:

            Independ.Claim Test.Type DF Crit.Value P.Value
     Buff.cor ~ KoBi + ...      coef 26    -0.1109  0.9125
  mean.height ~ KoBi + ...      coef 29    -1.3678  0.1819
       l.SSCI ~ KoBi + ...      coef 29     1.5580  0.1301
     Buff.cor ~ ScSu + ...      coef 26    -0.7881  0.4378
  mean.height ~ ScSu + ...      coef 29     1.2918  0.2066
       l.SSCI ~ ScSu + ...      coef 29    -0.1000  0.9210
     Buff.cor ~ QuFa + ...      coef 26     1.4561  0.1573
  mean.height ~ QuFa + ...      coef 29    -0.9980  0.3265
       l.SSCI ~ QuFa + ...      coef 29     1.0003  0.3254

--
Global goodness-of-fit:

Chi-Squared = 893.21 with P-value = 0 and on 9 degrees of freedom
Fisher's C = 20.824 with P-value = 0.288 and on 18 degrees of freedom

---
Coefficients:

       Response   Predictor Estimate Std.Error  DF Crit.Value P.Value
       Buff.cor mean.height  -0.0292    0.1357  22    -0.2152  0.8316
       Buff.cor      l.SSCI   0.1594    0.0611  22     2.6077  0.0161
       Buff.cor          BA   0.0262    0.0094  22     2.7788  0.0109
       Buff.cor   l.TreeDiv   0.0577    0.0729  22     0.7913  0.4372
         l.SSCI   l.TreeDiv   0.1712    0.0551  72     3.1062  0.0027
             BA   l.TreeDiv   3.0835    1.4933  27     2.0648  0.0487
             BA        KoBi -12.5003    4.9523  27    -2.5242  0.0178
             BA        ScSu  20.3708    5.4682  27     3.7253  0.0009
             BA        QuFa -12.1347    5.4682  27    -2.2191  0.0351
    mean.height   l.TreeDiv  -3.0058   30.2082  30    -0.0995  0.9214
           ~~BA    ~~l.SSCI   0.0003         - 192     0.0047  0.4981
  ~~mean.height    ~~l.SSCI  -0.1892         -  74    -1.6233  0.0545
  ~~mean.height        ~~BA  -0.0158         - 192    -0.2175  0.4140
```

```
  Std.Estimate
       -6.1221
        0.1069   *
        0.3568   *
        0.0731
        0.3236  **
        0.2873   *
       -0.3391   *
        0.5525 ***
       -0.3291   *
       -0.0182
        0.0003
       -0.1892
       -0.0158

  Signif. codes:  0 '***' 0.001 '**' 0.01 '*' 0.05

---
Individual R-squared:

     Response method Marginal Conditional
     Buff.cor   none     0.17        0.91
       l.SSCI   none     0.12        0.89
           BA   none     0.32        0.71
  mean.height   none     0.00        0.88
```
